# The Power of Catalytic Centers and Ascorbate in Boosting the Photocatalytic Hydrogen Evolution Performance of TpDTz 2D-COF

**DOI:** 10.1021/jacs.5c17806

**Published:** 2025-12-22

**Authors:** David Reyes-Mesa, Pau Sarró, Muriel F. Gusta, Alberto Jiménez-Solano, Saunak Das, Bishnu P. Biswal, Hugo A. Vignolo-González, Laura Velasco-Garcia, Antoni Llobet, Neus G. Bastús, Víctor Puntes, Adelina Vallribera, Roser Pleixats, Albert Granados, Bettina V. Lotsch, Carolina Gimbert-Suriñach

**Affiliations:** † Department of Chemistry and Centro de Innovación en Química Avanzada (ORFEO−CINQA), 16719 Universitat Autònoma de Barcelona, Cerdanyola del Vallès, Barcelona 08193, Spain; ‡ Catalan Institute of Nanoscience & Nanotechnology - ICN2 (BIST and CSIC), Campus UAB, 08193 Bellaterra, Barcelona, Spain; § Departamento de Física, 16735 Universidad de Córdoba, Edificio Einstein (C2), Campus de Rabanales, 14071 Córdoba, Spain; ∥ Nanochemistry Department, 28326 Max Planck Institute for Solid State Research, 70569 Stuttgart, Germany; ⊥ School of Chemical Sciences, 193155 National Institute of Science Education and Research (NISER), Khurda 752050 Odisha, India; # Homi Bhaba National Institute (HBNI), Training School Complex, Mumbai 400094, India; ∇ Institute of Chemical Research of Catalonia (ICIQ), Avda. Països Catalans 16, 43007 Tarragona, Spain; ○ Department of Chemistry, University of Stuttgart, 70569 Stuttgart, Germany; ¶ Department of Chemistry, University of Munich (LMU), 81377 Munich, Germany

## Abstract

The photocatalytic hydrogen evolution activity of a model 2D covalent organic framework (TpDTz) containing a thiazolo­[5,4-*d*]­thiazole (DTz) electron acceptor and triformylphloroglucinol (Tp) electron donor groups is enhanced by combining it with well-defined catalytic centers and suitable sacrificial electron donors. Platinum nanoparticles (PtNPs) with an average diameter of 2.7 ± 0.4 nm achieve rates up to 106 000 μmol H_2_ g^–1^ h^–1^ (5% Pt w/w). The best system requires the use of ascorbic acid/ascorbate buffer, which has been demonstrated to enhance the photoluminescence of TpDTz by forming aggregates while efficiently extracting charges from the excited TpDTz (TpDTz*). The productive charge extraction by the PtNPs from TpDTz* is also supported by steady state and time-resolved photoluminescence studies. All these factors combined with the high catalytic activity of PtNPs catalytic centers lead to the high performance of the overall system. In addition, a noble metal-free molecular catalyst based on a tetraazamacrocyclic cobalt complex has been identified as a good alternative catalyst candidate, efficiently quenching TpDTz photoluminescence. Under optimal conditions, the cobalt-based system achieves catalytic rates of 10 400 μmol H_2_ g^–1^ h^–1^ (1% Co w/w)_,_ which is only three times slower than the noble metal-based PtNPs system (1% Pt w/w, 28 300 μmol H_2_ g^–1^ h^–1^). By using controlled catalytic centers, it was possible to identify the factors limiting the hydrogen evolution photocatalytic activity of TpDTz allowing one to minimize undesired pathways and enhancing its performance by 2 orders of magnitude.

## Graphical abstract

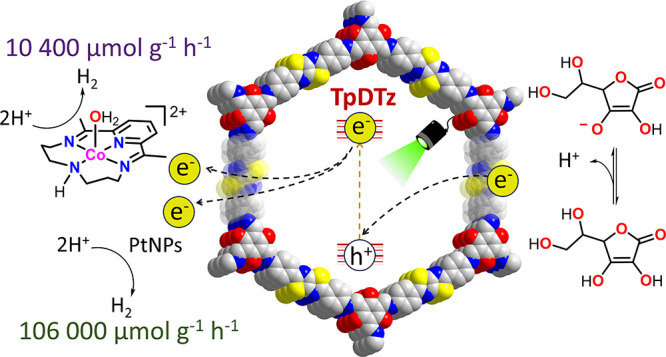

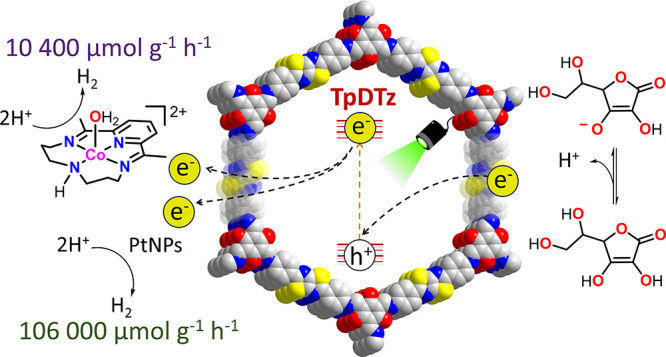

## 1 Introduction

Covalent organic frameworks (COFs) have become powerful organic photoactive materials for the light-induced hydrogen evolution reaction (*hυ*-HER) in aqueous media.^1–5^ In particular, two-dimensional materials (2D-COF) made from rigid and/or planar polyfunctional monomeric precursors have shown impressive catalytic rates that have reached turnover frequencies in the hundreds of thousands of μmol H_2_ g^–1^ h^–1^,^6–8^ values that are 4 orders of magnitude higher than that obtained for the first reported *hυ*-HER with a 2D-COF ten years ago.^9^ These materials are characterized by their high degree of tunability, which allows rational change of their properties by carefully selecting the precursors that build their structure. Thus, several studies looking at structure–function relationships have found the important role of a series of factors that are key to understanding such significant improvements in catalytic rates and efficiency. For instance, the optical and electronic properties of 2D-COF have been significantly improved by choosing chromophore moieties and adding electron donor together with electron acceptor groups.^10,11^ Controlling crystallinity as well as pore or crystallite size allows to increase the surface area of these catalytic materials^12–16^ while polar groups are crucial to increase their hydrophilicity.^17^

Another important parameter influencing the *hυ*-HER catalytic performance of 2D-COF is the use of catalysts. While the organic material is primarily responsible for light absorption, the catalyst is in charge of the chemical conversion, *i.e.* bond breaking and bond formation, far more efficiently than the pristine COF would do. Both the COF material and the catalyst must operate in synchrony to enable charge separation followed by charge transfer, ultimately leading to hydrogen evolution from water. Current photocatalytic systems mostly rely on using platinum catalytic centers (mainly nanoparticles) that are photodeposited *in situ* from hexachloroplatinic acid or its derivative salts.^18–20^ Although such an approach has led to incredibly fast systems, these systems lack control over the formation of the real photocatalytic species, formed under catalytic turnover. Sometimes even traces of palladium originating from cross-coupling reactions involved in the synthesis of similar materials such as covalent triazine frameworks materials have been demonstrated to be responsible for their HER catalytic performance without the need of an additional catalyst.^21^ More recently, less expensive metals such as cobalt or nickel have been used and achieved remarkable results.^22–26^

In this work, we study the role of hydrogen evolution catalytic species in *hυ*-HER by using a model 2D-COF photoabsorber. With this aim, we combine the 2D-COF with discrete molecules or preformed metallic nanoparticles as catalysts, allowing us to decouple the two components of the photocatalytic system (photoabsorber and catalytic center) and identifying key factors that boost the catalytic activity toward benchmarking rates.

## 2 Results and Discussion

### 2.1 Photocatalytic System Selection and Preparation

The electron donor–acceptor nature of the 2D-COF structures is key to increase light absorption, facilitate charge separation, and ultimately allow charge transfer to the catalyst for HER catalysis. The TpDTz material in Figure 1a is an enamine linked 2D-COF constituted by thiazolo­[5,4-*d*]­thiazole electron acceptor groups^27,28^ directly attached to electron rich aromatic rings with an optical band gap of 2.1 eV. It has shown good HER performance in the presence of a catalyst formed *in situ* from Ni salts and mercaptoetanol.^24^ Interestingly, the same material appears to be inactive in the presence of Pt nanoparticles generated *in situ* from H_2_PtCl_6_ under analogous conditions. Taking advantage of the electronic properties and promising HER catalytic activity, TpDTz was selected as a model photoactive material for this study. The material was successfully prepared in batches exceeding 500 mg, following a metal-free synthetic pathway. This new synthetic route avoids a highly insoluble dinitroderivative intermediate that was poorly reproduced and hindered the large scale production of the material (Scheme S1).^24^ The structural and morphological features of the synthesized TpDTz 2D-COF are consistent with earlier findings.^24^ Detailed characterization enabled by powder X-ray diffraction (PXRD, Figure S1), high-resolution transmission electron microscopy, scanning electron microscopy (HR-TEM and SEM, respectively, Figure S2) and Brunauer–Emmett–Teller analysis (BET, Figure S3) show the expected crystallinity and high surface area (721 ± 10 m^2^ g^–1^). Infrared (IR), diffuse reflectance ultraviolet–visible (DR UV–vis) and photoluminescence (PL) spectroscopies allowed us to fully characterize the chemical, optical, and photoluminescent characteristics of the synthesized material (Figure 1b and Figures S4, S6). TpDTz is characterized by a light absorption profile expanding beyond 600 nm with a broad maximum of light absorption in the range of λ_max_ = 440–550 nm and a prominent PL signal centered at λ_max_ = 616 nm as shown in the absorption and PL spectra, respectively, obtained for an aqueous 0.5 mg/mL suspension of the material (Figure 1). Cyclic voltammetry analysis of a FTO (fluorine doped tin oxide) electrode functionalized with TpDTz was used to estimate the conduction band (CB) and valence band (VB) position values, that agree with Density Functional Theory (DFT) calculations of a TpDTz pore model (Figure 1a and Figure S36).^24^

**Figure 1:**
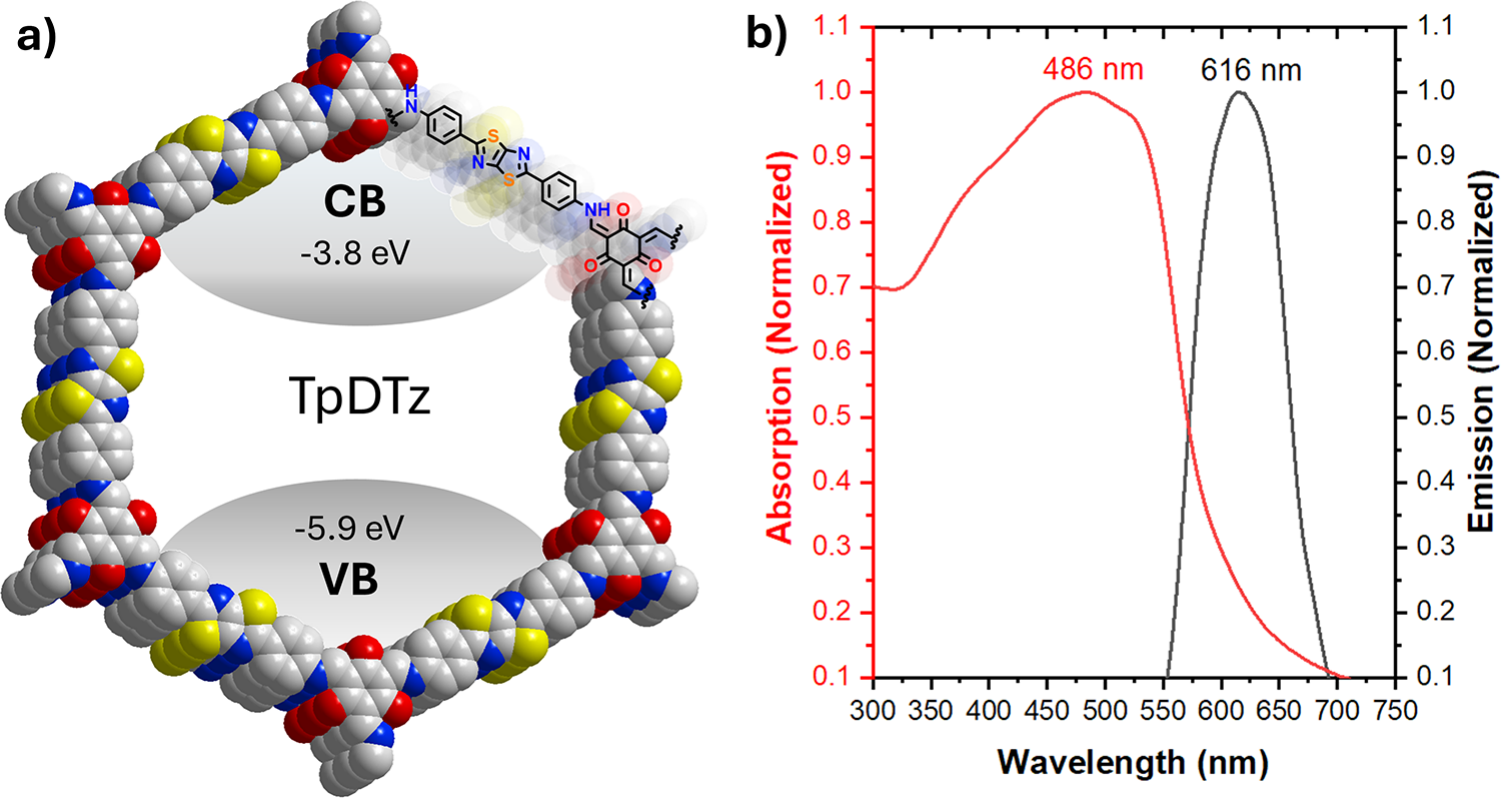
(a) TpDTz 2D-COF structure containing thiazolo­[5,4-*d*]­thiazole and phloroglucinol moieties with valence band (VB) and conduction band (CB) energies estimated from combined CV and optical band gap analysis, in agreement with DFT calculations.^24^ Color code: gray for carbon, red for oxygen, blue for nitrogen, and yellow for sulfur. (b) DR UV–vis absorption (red) and emission (black, λ_exc_ = 365 nm) spectra of a suspension of TpDTz in water (0.5 mg TpDTz mL^–1^).

Next, three well-defined catalytic centers were selected and compared with the catalytic species that were formed *in situ* in the previous work (Figure 2).^24^ First, monodisperse platinum nanoparticles (PtNPs) stabilized by citrate ions with an average diameter of 2.7 ± 0.4 nm were synthesized by the chemical aqueous reduction of potassium hexachloroplatinate (K_2_PtCl_6_) by sodium borohydride in the presence of sodium citrate (Figure S5).^29^ Platinum metal is the benchmark catalyst for the HER reaction, thanks to its intrinsic properties related to H atom/H^+^ absorption and H_2_ desorption energies.^30,31^ The use of small nanoparticulate species provides a high surface area and increased chances to be intercalated within the porous structure of the TpDTz material.

**Figure 2:**
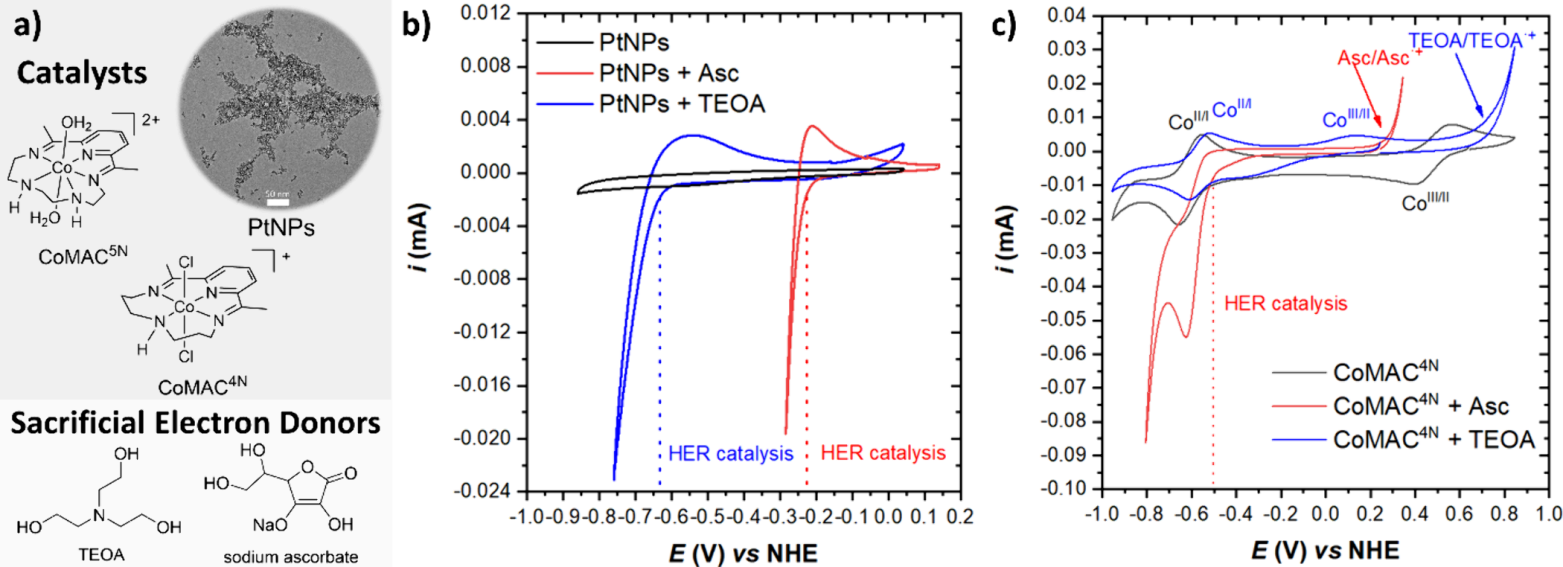
(a) Selected catalysts and sacrificial electron donors. (b) Cyclic voltammetry (CV) of PtNPs. (c) CV of CoMAC^4N^. For (b) and (c) experiments were run in water-0.1 M Na_2_SO_4_ (black traces) or in the presence of two sacrificial electron donors: a 1:1 mixture of ascorbic acid and sodium ascorbate (Asc, pH = 4.1, red traces) or triethanolamine (TEOA, pH 10.9, blue traces) using a glassy carbon disk WE (A = 7 mm^2^), Pt mesh as CE and SCE as RE (converted to NHE by adding 0.241 V).

The PtNPs were characterized by cyclic voltammetry in aqueous conditions, in the absence and presence of common sacrificial electron donors (Figure 2b and Figure S9). They show an onset of the HER catalysis at −0.64 V and −0.26 V vs NHE in the presence of triethanolamine (water:TEOA 9:1, pH 10.9) or in ascorbic acid/sodium ascorbate buffer (pH 4.1), respectively. This difference in potential and pH can play a key role when the PtNPs catalyst is combined with the 2D-COF photoabsorber, determining driving forces for charge transfer events and HER catalytic rates that should all synchronize. Importantly, cyclic voltammetry analyses of preformed PtNPs in Figure 2b confirm that the electron transfer from the CB of TpDTz to the PtNPs is thermodynamically favored, thus allowing the activation of the PtNPs catalyst that will trigger the proton to hydrogen conversion.

In addition, two cobalt complexes containing tetraaza- or pentaaza-macrocyclic ligands were chosen, named CoMAC^4N^ and CoMAC^5N^, respectively (Figure 2a). They were selected for their well-known HER catalytic activity and enhanced stability as compared to other molecular catalysts.^32–41^ Both complexes were characterized by cyclic voltammetry, showing catalytic onset potentials at −0.50 V vs NHE (CoMAC^4N^) and −0.68 V vs NHE (CoMAC^5N^) in ascorbic acid/sodium ascorbate buffer (pH 4.1, red trace in Figure 2c and Figures S10–S12). On the other hand, they show no significant electrocatalytic behavior in the presence of triethanolamine (pH 10.9, blue trace in Figure 2c and Figures S10–S12). Although the HER overpotentials required for CoMAC^4N^ or CoMAC^5N^ are higher than the PtNPs overpotential in ascorbate buffer, a thermodynamically favored electron transfer from the TpDTz CB is still viable, setting both cobalt catalysts as good candidates for the multicomponent photocatalytic system.

### 2.2 The Influence of Catalytic Centers and SED on Light-Induced HER

The original work on TpDTz as a photoabsorbing material for the *hυ*-HER reaction resulted in practically null activity of the system when using H_2_PtCl_6_ as the catalyst precursor, although the presence of metallic nanoparticles within the COF structure were found in HR-TEM images of the mixture after illumination.^24^ Intrigued by this result, an analogous experiment was performed using TpDTz with a concentration of 0.5 mg·mL^–1^ in a mixture of water:TEOA 9:1, and containing 5% (w/w) of the presynthesized PtNPs shown in Figure 2a and we did not detect any hydrogen formation. Importantly, the original catalytic conditions reproduced here use a basic unbuffered solution containing TEOA as sacrificial electron donor (SED) agent, a popular SED that has resulted in very convenient and efficient catalytic performance when using other 2D-COFs in combination with platinum based catalysts (Table S2).^5,42,43^ Indeed, the cyclic voltammetry analysis of the PtNPs in the presence of water:TEOA = 9:1 (pH 10.9) shows a significant catalytic performance with an onset of the HER catalysis at −0.63 V vs NHE, which accounts for an overpotential of only η = 19 mV (Figure 2b, blue trace). The pH of the solution does not have a large influence on the catalytic rate of the PtNPs obtaining comparable slopes of the catalytic waves at pH 10.9 and 4.1 (Figure S9). These results suggest that the HER performance of the system may not be limited by platinum intrinsic activity at pH 10.9 but hindered by inefficient charge transfer events between the three components (PtNPs/TpDTz/TEOA).

To get more insights into the limiting factors of our catalytic systems, the experiments were repeated using an ascorbic acid/sodium ascorbate buffer solution as the SED (Figure 3a). The systems showed excellent catalytic activity with rates of 22 000 μmol H_2_ g^–1^ h^–1^ and 15 900 μmol H_2_ g^–1^ h^–1^ using H_2_PtCl_6_ as catalyst precursor or presynthesized PtNPs, respectively.

**Figure 3:**
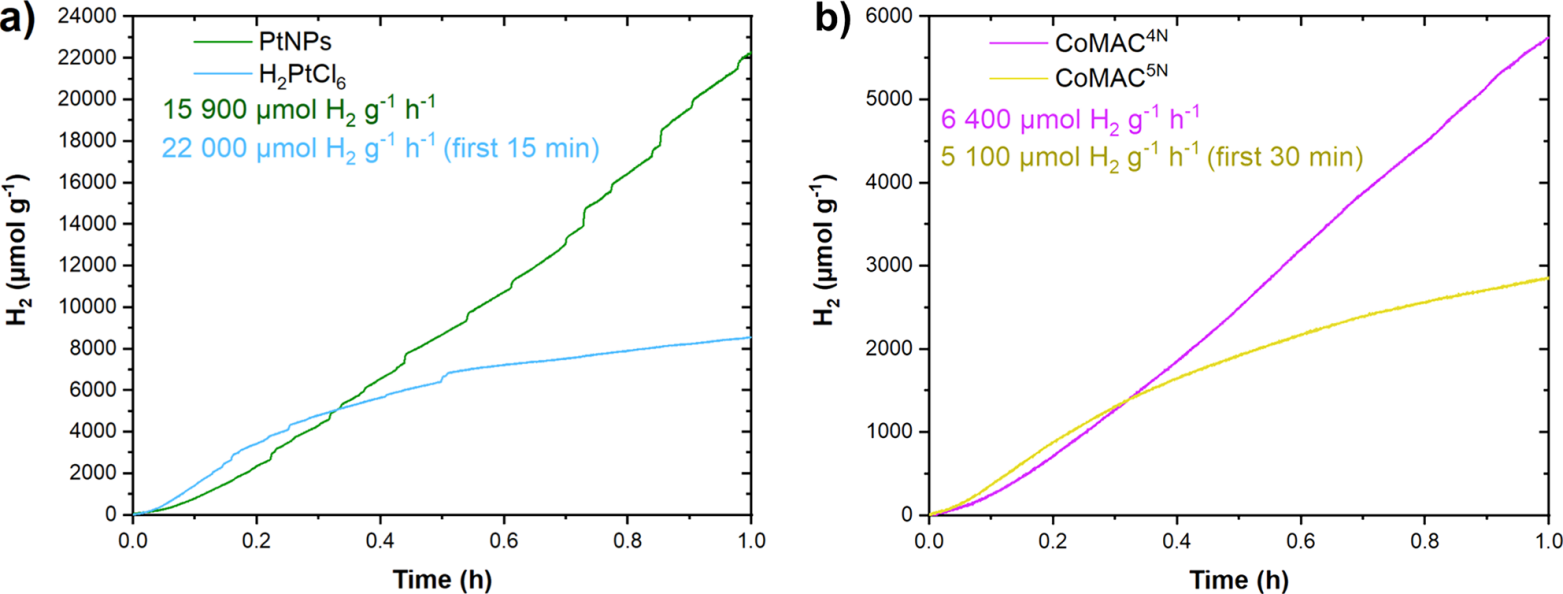
Comparison of light-induced hydrogen evolution reaction using TpDTz (0.5 mg mL^–1^) and different catalysts in the presence of ascorbic acid/sodium ascorbate (Asc, 1.6 M) SED under white light illumination AM 1.5G (100 mW cm^–2^). (a) System based on PtNPs (5% Pt w/w) formed *in situ* (blue) or presynthesized (green). (b) System based on cobalt molecular complexes (1 mM, 1% Co w/w) CoMAC^4N^ (purple) or CoMAC^5N^ (yellow).

These results clearly indicate that the performance of the TpDTz/PtNPs photocatalytic system is highly dependent on the nature of the SED, as it governs both the pH of the catalytic solution and the driving force for charge transfer between TpDTz and the SED. Interestingly, the system that uses H_2_PtCl_6_ shows superior performance at lower reaction times, but it deactivates very fast, reducing its activity to about 8 600 μmol H_2_ g^–1^ h^–1^ after 15 min and is less reproducible (see Figures S21 and S25). This is a consequence of the *in situ* formation of the catalyst from H_2_PtCl_6_ under catalytic conditions and highlights the advantage of using presynthesized PtNPs. Indeed, the growth of the NPs in such heterogeneous media is highly dependent on several factors resulting in poor control of size and homogeneity of the formed NPs, including concentration of SED (contributing to the stabilization of NPs), reducing power of SED (involved in Pt­(IV) reduction), pH, reaction time, homogeneity of colloidal mixture, etc. In sharp contrast, the system composed of presynthesized PtNPs is still active after one h with constant catalytic rate, highlighting the controlled nature of the catalyst species.

Next, noble metal-free catalysts CoMAC^4N^ and CoMAC^5N^ were tested in combination with TpDTz in analogous catalytic conditions based on the ascorbic acid/sodium ascorbate buffered mixture. As shown in Figure 3b, both catalysts are active, achieving remarkable turnover frequencies of 6 400 μmol H_2_ g^–1^ h^–1^ and 5 100 μmol H_2_ g^–1^ h^–1^ for CoMAC^4N^ and CoMAC^5N^, respectively. While the catalytic activity of the system based on CoMAC^5N^ deactivates significantly after 30 min of photoirradiation (yellow trace), that based on CoMAC^4N^ is still fully operative after one hour (purple trace). Increasing the concentration of CoMAC^4N^ led to a reduction in catalytic performance, a phenomenon that we attribute to the light absorption capacity of the Co­(II) derivative formed when CoMAC^4N^ was mixed with the ascorbic acid/ascorbate buffer (Figures S8 and S23). The broad range absorption of light by this catalytic species may partially hinder the photoactivation of the TpDTz material and contribute to a competitive energy transfer phenomenon (*vide infra*).

It is important to highlight that different pHs provided by the two SED have an impact on the operating potential and rate in which the catalyst can perform the HER. This is a consequence of the favored thermodynamics of the HER at low pHs but also the mechanistic pathways leading to bond formation-breaking. This is clearly reflected by the cyclic voltammetry analysis of the catalysts (Figure 2 and Figures S9–S12). The faster the chemical reaction, the lower the chance of charge recombination, favoring the overall reaction. While the pH is not a determining factor for the PtNPs catalyst rate (Figure 2b and Figure S9), it is for the cobalt-based catalysts, both showing higher performances under buffered acidic conditions (compare red and blue traces in Figure 2c, and Figures S10, S11). In particular, the operating mechanism of CoMAC^4N^ has been previously studied, proving its limited catalytic rates at pH > 4.1 due to the slow formation of the Co­(III) hydride species being the rate limiting step at higher pHs.^37^ Interestingly, the related CoMAC^5N^ catalyst shows a superior catalytic activity under electrocatalytic conditions, but requires a significantly lower operating potential (Figure S12).

### 2.3 Enhancing the Photocatalytic Activity by Focusing on TpDTz Excitation

Light-induced HER experiments are usually run under standardized illumination, which involves the use of solar simulators, at an irradiation power of 100 mW·cm^–2^. However, such broad-spectrum light sources may be detrimental for certain photocatalytic reactions, particularly in *hυ*-HER by 2D-COFs, which usually involve multicomponent systems leading to multiple excited species. For instance, the DR UV–vis spectrum of a colloidal suspension containing TpDTz 0.5 mg·mL^–1^ shows a broad maximum of the light absorption in the range of λ_max_ = 440–550 nm (Figure 1b) while the UV–vis spectrum of the presynthesized PtNPs shows a featureless absorption profile with increasing extinction coefficients toward λ < 300 nm (Figure S7). The cobalt complexes also show significant light absorption with the most intense bands laying at λ < 350 nm although they also present bands in the visible light range with lower extinction coefficients (Figure S8). To optimize the utilization of the absorbed photons in our catalytic systems, we repeated the whole set of catalytic combinations but using a LED light focused on the maximum absorption band of the TpDTz material (λ_em_ = 525 nm, 100 mW·cm^–2^).

As shown in Figure 4, the catalytic performance of platinum-based catalysts improves when using LED light achieving rates of 106 000 μmol H_2_ g^–1^ h^–1^ and 79 600 μmol H_2_ g^–1^ h^–1^ using presynthesized PtNPs or H_2_PtCl_6_ as catalyst precursor, respectively. In the case of the presynthesized PtNPs the catalytic rate increases by about seven times compared to the performance observed under white light illumination (Figure 3a), highlighting the beneficial role of centering the excitation to the maximum absorption of the TpDTz photocatalyst. Such catalytic activity using 2D-COF has been achieved with limited examples including COF-JLU100,^5^ TFP-BpyD nano-COF,^6^ COF-JLU45,^7^ Ni-COF-SCAU-1,^8^ COF-954,^44^ FOOCOF-PDI,^45^ or TpPa-SCOF-An.^46^ Other related systems with remarkable catalytic activity are carbon nitrides,^47^ conjugated polymers,^48^ molecular organic dyes^49^ or organic materials formed by supramolecular assemblies.^50,51^ A comparison of turnover frequencies, catalytic species, sacrificial electron donor, and light sources for these benchmarking systems is given in Table S2. They all use Pt(0) as the catalyst generated *in situ* from H_2_PtCl_6_ in the range of 0.7–15% w/w. In the set of experiments summarized in Figure 4, the system based on presynthesized PtNPs is superior over the whole reaction time, a result associated with the lack of UV light that cannot contribute to the photoreduction of the H_2_PtCl_6_ precursor, thus hindering the formation of the real nanoparticulate catalyst.^52^ Under LED light irradiation, the reduction of Pt­(IV) necessary to form the Pt(0) nanoparticles relies uniquely on the reducing power of the ascorbate and the excited or photoreduced TpDTz material. The change in light source also translates into the higher stability of the H_2_PtCl_6_ catalysis that remains active after 1 h of experiment, but the system is less reproducible than that based on presynthesized PtNPs, as expected for an uncontrolled *in situ* formation of the catalyst.

**Figure 4:**
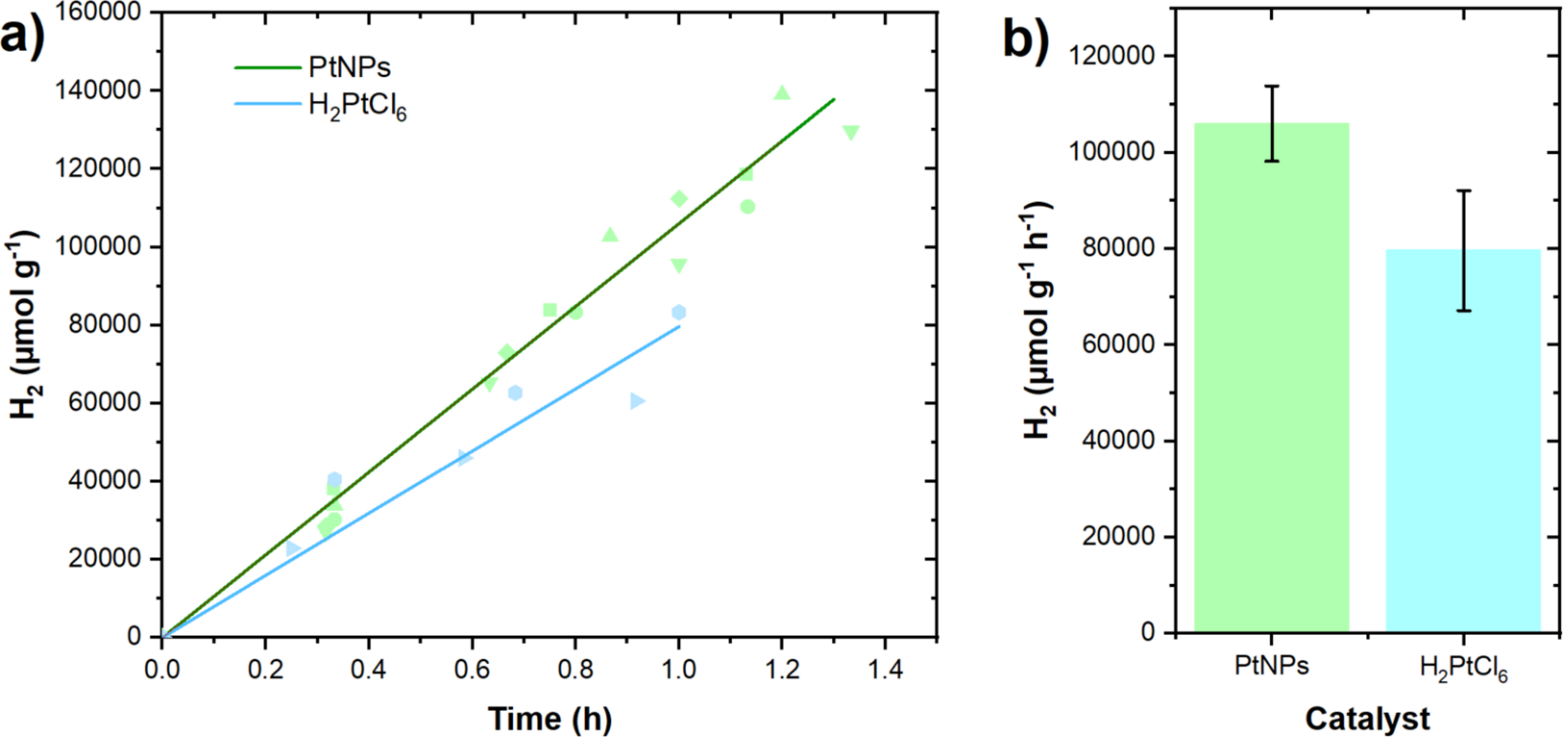
Light-induced hydrogen evolution reaction using TpDTz (0.5 mg mL^–1^) and platinum nanoparticles catalyst formed *in situ* (blue) or presynthesized (green) at 5% of metal (w/w) in the presence of ascorbic acid/sodium ascorbate (Asc, 1.6 M) sacrificial under LED light illumination (λ_em_ = 525 nm, 100 mW cm^–2^). (a) Hydrogen evolution profile over time. (b) Comparison of turnover frequencies for the two systems.

The performance of the cobalt-based systems also improves significantly under LED irradiation (Figure 5). Remarkable catalytic rates of 10 400 μmol H_2_ g^–1^ h^–1^ and 6 500 μmol H_2_ g^–1^ h^–1^ are achieved for CoMAC^4N^ and CoMAC^5N^, respectively, which are on the same order of magnitude as that of the PtNPs system when the same loading of metal catalyst is used (1% w/w). Encouraged by these results using non-noble metal catalysts, we revisited the nickel catalyst previously reported achieving activities of 2 400 μmol H_2_ g^–1^ h^–1^, significantly higher than the performance obtained in our original work using a solar simulator light source (941 μmol H_2_ g^–1^ h^–1^, Table S2).^24^ As shown in Table S2 (gray-shaded), only a few examples of 2D-COFs employing non-noble metal catalysts exhibit moderate catalytic activity, positioning CoMAC^4N^/TpDTz/Asc and CoMAC^5N^/TpDTz/Asc among the most efficient platinum-free hydrogen evolution photocatalytic systems based on 2D-COFs.

**Figure 5:**
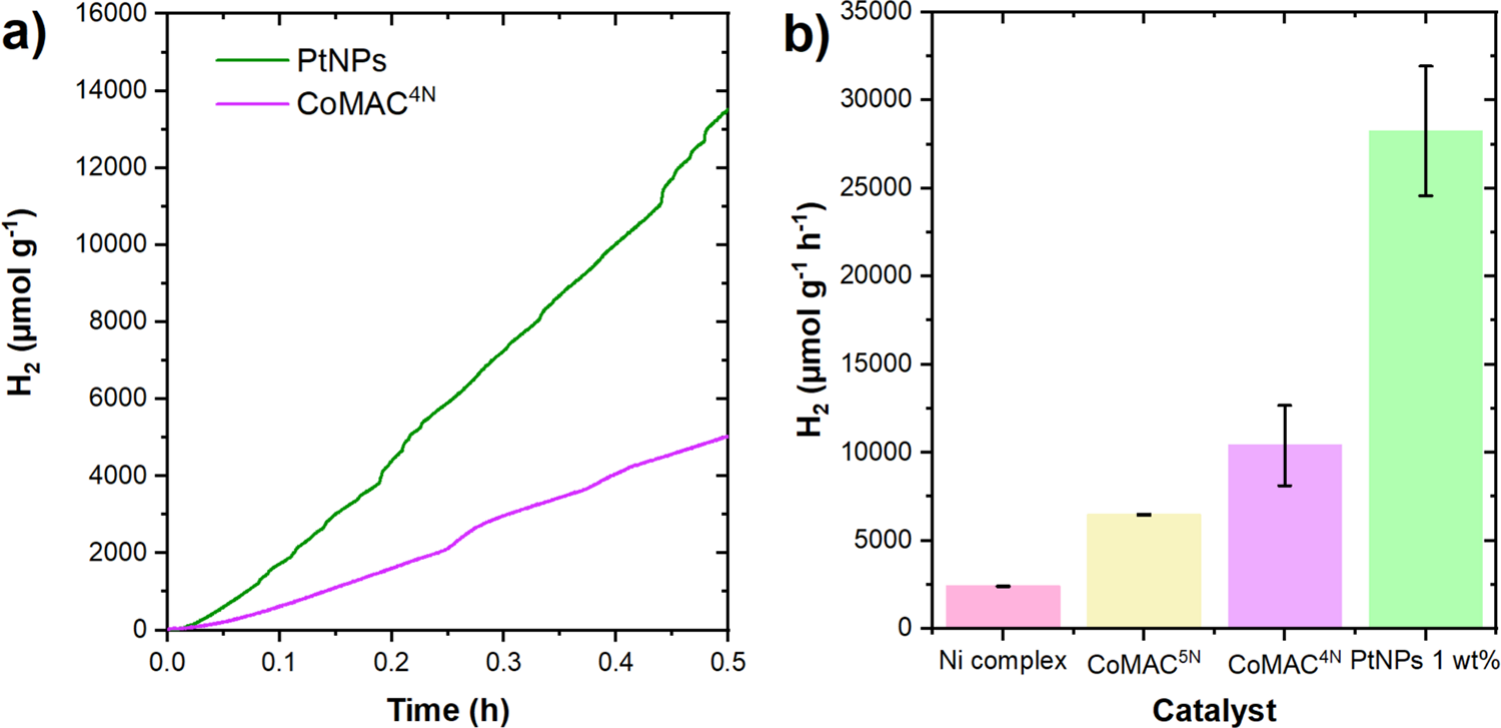
Light-induced hydrogen evolution reaction using TpDTz (0.5 mg·mL^–1^) in the presence of ascorbic acid/sodium ascorbate (Asc, 1.6 M) and different catalysts at 1% of metal (w/w) under LED light illumination (λ_em_ = 525 nm, 100 mW·cm^–2^). For the Ni complex, 2.4% of metal was used in combination with TEOA SED (pH = 10.9) for comparison purposes with previous work.^24^ (a) Hydrogen evolution profile over time for the best catalysts, PtNPs (green) and CoMAC^4N^ (purple). (b) Comparison of turnover frequencies for different catalytic systems.

Blank experiments, run in the absence of TpDTz, catalyst, or light, demonstrate that all components are necessary to achieve the catalytic process (Figures S27). Postcatalysis analyses were carried out to gain insight into the structural features of the photoactive material after turnover. Thus, after 1 h of photocatalysis, the supernatant was separated by centrifugation, the remaining solid material was washed twice with MeOH and once with DCM solvent and analyzed by HR-TEM and PXRD. HR-TEM analysis of the PtNPs/TpDTz system shows that the nanoparticles remain attached to the organic material and that they maintain their size with a calculated average diameter of 3.1 ± 0.5 nm (Figure S30), forming hybrid material TpDTz@PtNPs. This result suggests an efficient nanoparticle ligand exchange taking place with the 2D-COF,^53^ presumably favored by the porous nature of the material as well as its conjugated chemical structure, rich in heteroatoms. Importantly, this hybrid material TpDTz@PtNPs is responsible for the fast catalysis as demonstrated by running a second catalytic cycle after separating and washing the solid and without adding additional PtNPs (Figure S28). The intercalation of PtNPs within the COF structure results in some loss of crystallinity, although some crystalline domains are still observed in the material after catalysis (Figure S35). On the other hand, HR-TEM analyses of catalytic mixtures containing H_2_PtCl_6_ confirm the *in situ* formation of nanoparticles with a slightly smaller size (2.5 nm), higher polydispersibility and a lack of reproducibility (Figures S32). This is a consequence of the poor control of the parameters influencing the nanoparticles’ growth in the complex catalytic mixture. In the case of CoMAC^4N^ or CoMAC^5N^, no metal content could be detected by EDX analysis (Figures S33, 34), suggesting a weak interaction with the organic material that can be broken during the washing steps.

### 2.4 Insights into Charge Transfer Processes through PL and TCSPC Experiments

The catalytic results discussed above reflect the importance of choosing the right catalyst and SED combination to get the maximum outcome of the photocatalytic system, a common observation in multicomponent photocatalytic systems.^17,54–56^ This is a consequence of the array of steps required to perform light-induced hydrogen evolution catalysis, which requires precise synchronization of the processes involving interactions of the components in the mixture. Thus, the TpDTz material should have a feasible electron transfer pathway based on matching the energy level alignment with both the SED and the catalytic species. In addition, the charge transfer events should be fast enough to allow the formation of the H_2_ molecule before charge recombination or undesired side-reactions occur. These processes are often difficult to study when the catalyst is formed *in situ* during the catalysis due to the difficulty in decoupling the light absorber and the catalytic center where water is transformed into H_2_. Taking advantage of the decoupled-multicomponent nature of our catalytic system as well as the fluorescence of the TpDTz material, we sought insights into the charge transfer processes by means of photoluminescence (PL) quenching experiments. As shown in Figures 6 and Figures S13, S14, TpDTz shows a weak but prominent PL signal centered at λ_max_ = 616 nm in aqueous solution after excitation at λ_exc_ = 500 nm, simulating the light source of the optimized photocatalytic experiments. This PL is significantly quenched by PtNPs or CoMAC^4N^ leaving only 56% and 81% of the original emission after adding 5% of the quencher, respectively (Figure 6a and 6b, respectively).

**Figure 6:**
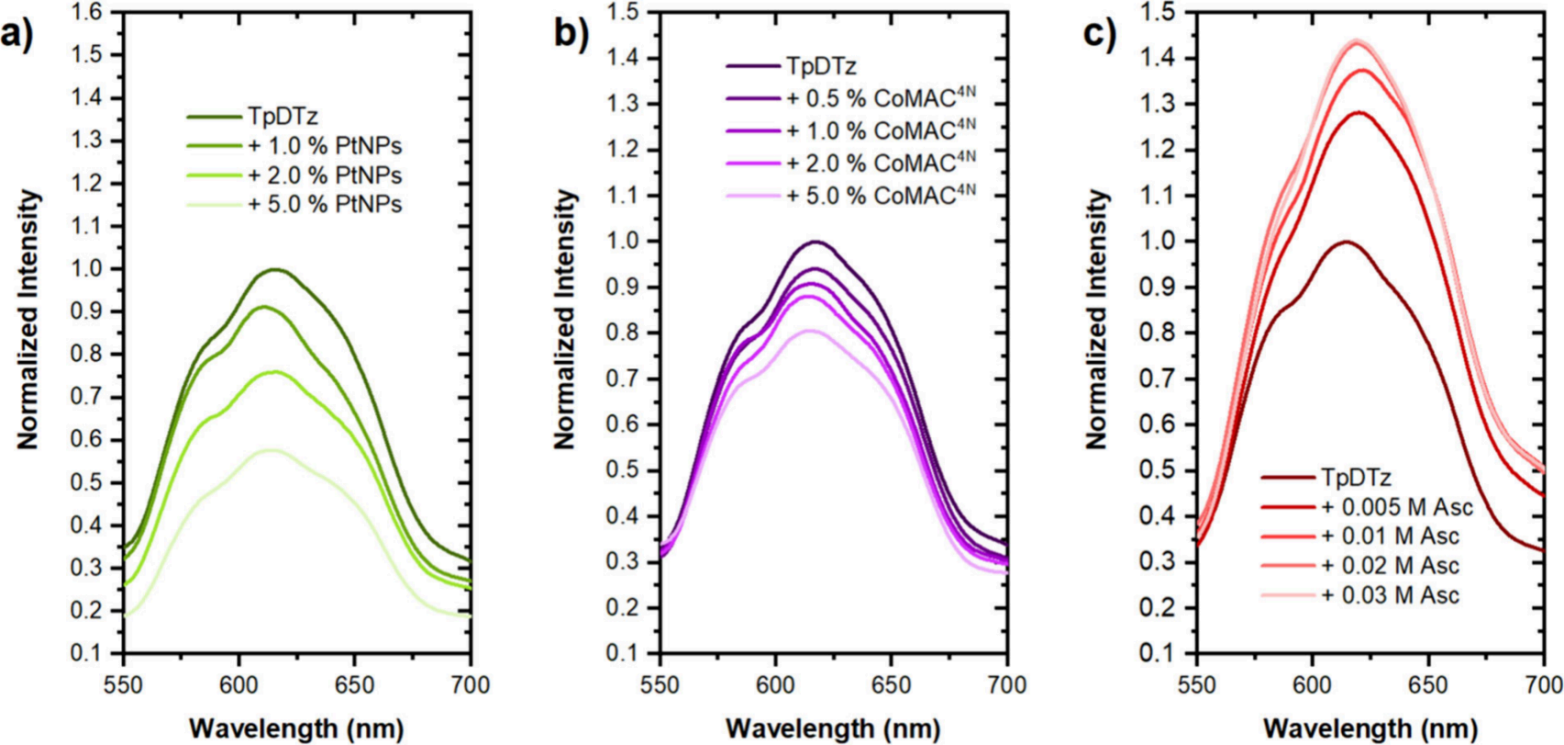
Photoluminescence quenching experiments of an aqueous solution of TpDTz (0.5 mg of TpDTz mL^–1^) in the presence of different quenchers (λ_exc_ = 500 nm). (a) PtNPs (1–5% w/w). (b) CoMAC^4N^ (0.5–5% w/w). (c) 1:1 mixture of ascorbic acid/sodium ascorbate (Asc) (0.005–0.03 M).

Among the different phenomena that can lead to PL quenching, we propose an electron transfer (ET) from the excited TpDTz (TpDTz*) to the catalytic species, a key process in the overall hydrogen evolution reaction (Scheme 1, ET from the conduction band of TpDTz to catalyst PtNPs or CoMAC^4N^). To shed more light on this phenomenon and help distinguish between the possible mechanisms involved in PL quenching, time-resolved PL measurements were performed and are discussed below. Interestingly, when a 1:1 mixture of ascorbic acid/sodium ascorbate (Asc) is added, an increase of the emission of the TpDTz is observed (Figure 6c). While this PL enhancement phenomenon hinders our analysis of the electron transfer process, it provides useful information on the additional role of Asc in the photocatalysis experiment beyond acting as SED. Indeed, when Asc is added into a TpDTz suspension, a macroscopic aggregation of the material is observed (Figure S15), suggesting that the emission at λ_max_ = 616 nm may be enhanced by an aggregated state of the organic material. In addition, Asc changes the pH of the medium presumably inducing partial protonation of the TpDTz framework, leading to a different electronic configuration of the conjugated structure.^57–59^ This is supported by a slight red shift of the DR UV–vis spectrum in the solid state (Figure S15). Finally, Asc can form aggregates that might contribute to additional TpDTz excitation through scattering or clusteroluminescence resulting in enhanced PL in our binary Asc/TpDTz mixture.^60^ All of these phenomena are not observed with TEOA, which shows negligible quenching of the TpDTz PL and no macroscopic aggregation (Figure S14). Importantly, mixtures containing the three components Asc/TpDTz/catalyst for the best catalytic systems (PtNPs and CoMAC^4N^) show a significant quenching of the original PL spectrum, suggesting that even if the Asc contributes to an enhanced PL, the catalytic species can efficiently extract the generated charges of TpDTz*. In particular, the ternary mixture Asc/TpDTz/CoMAC^4N^ shows a remarkable 48% PL quenching (Figure S13), much higher than that observed for the binary mixture TpDTz/CoMAC^4N^ (19% PL quenching, Figure 6b). In the presence of Asc, the CoMAC^4N^ in the Co­(III) state is reduced to Co­(II) thanks to the reducing power of ascorbate (Figure S8).^34,36^ Thus, in the ternary mixture experiment, we are assessing the electron transfer from TpDTz* to the cobalt, which converts Co­(II) to Co­(I). Instead, in the absence of ascorbate (binary mixture TpDTz/CoMAC^4N^), the analogous electron transfer is responsible for the initial Co­(III) to Co­(II). Both conversions are key in the overall catalytic mechanism by CoMAC^4N^ illustrated in Scheme S3, proposed based on previous reports together with the results described here.^36,37^ Thus, two electron transfers are necessary to obtain the Co­(I) active species that reacts with H^+^ to generate the key Co­(III) hydride intermediate that will lead to H_2_ evolution. In addition, energy transfer from TpDTz* to CoMAC^4N^ is also plausible in the ternary Asc/TpDTz/CoMAC^4N^ mixture as suggested by the extended absorption of CoMAC^4N^ in the Co­(II) state compared to the Co­(III) state and supported by the shift in the maximum of the emission peak from λ_max_ = 616 nm to λ_max_ = 636 nm (Figures S8 and S13).

**Scheme 1:**
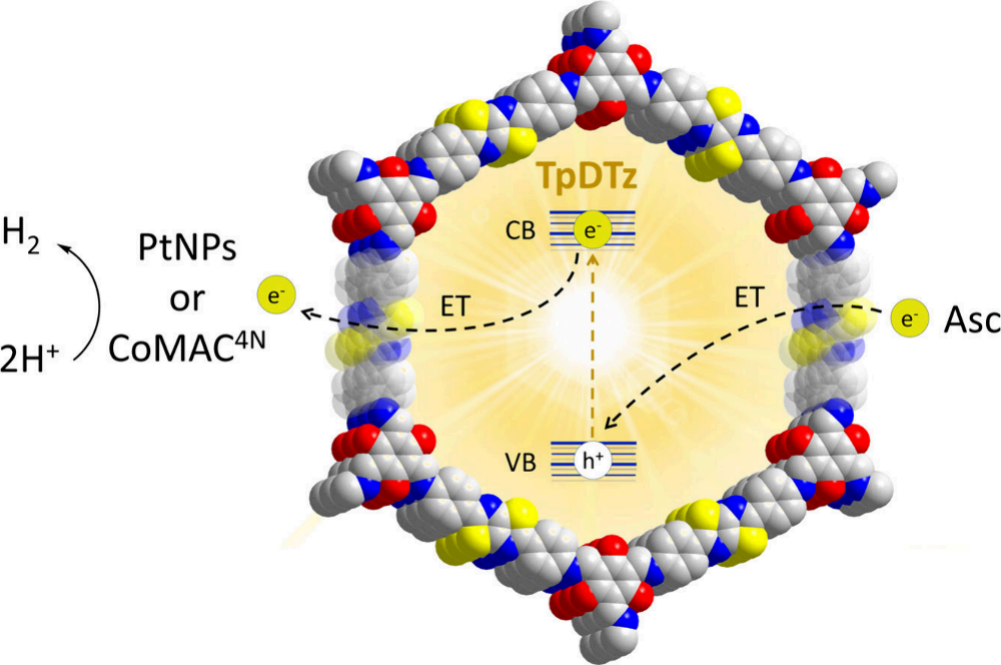
Electron Flow of the Multicomponent System Composed by TpDTz Acting as the Light Harvester, Catalyst PtNPs or CoMAC^4N^ Being Responsible for Charge Separation and Hydrogen Evolution Catalytic Transformation and Ascorbic Acid/Ascorbate (Asc) as the Sacrificial Electron Donor^a^

a VB: valence band. CB: conduction band. ET: electron transfer.

Time Correlated Single Photon Counting (TCSPC) experiments of analogous mixtures allowed us to interrogate radiative and nonradiative phenomena taking place when different components are mixed (Figure 7). The TCSPC signal could not be fitted with a single exponential function, even for the simplest unicomponent case, composed of a suspension of the TpDTz particles. This is due to different factors, including the intrinsic complex electronics of 2D-COF materials and the polydisperse nature of the TpDTz synthesized in this work, with different crystallite sizes and heterogeneous morphology. All together may lead to distinct surface recombination processes and scattering behavior.^8,61–63^ Instead, the data were successfully fitted by a combination of an exponential and a Γ-function leading to two decay lifetimes (τ_exp_ and τ_mean_, respectively), both contributing to the overall lifetime (τ_av_) with different weight factors (Figures S17–S19 and Table S1). Minor features attributed to secondary reflections (∼1.3 ns) are visible in both the decay traces and the experimentally measured Instrument Response Function (IRF). Importantly, the lifetime analysis was performed through convolution fitting using the measured IRF, without fitting it. This approach ensures that instrumental artifacts, including secondary reflections, are accurately accounted for in the fitting procedure, thereby minimizing their influence on the extracted decay times. The unicomponent TpDTz suspension shows an average value of 0.46 ns (±0.05 ns) estimated from independent samples, an extremely fast decay indicative of multiple intrinsic recombination pathways within the covalent organic framework before adding any additional component. When PtNPs are added, the average lifetime is 15% shorter (Figure 7a). These results agree with the PL quenching experiments in Figure 6 and support the extraction of excited electrons in TpDTz* by the PtNPs, a key process in the overall catalytic process (Scheme 1, ET from the conduction band of TpDTz to PtNPs). The lifetime is reduced to 59% when Asc is added, further supporting the role of Asc in extracting charges (Scheme 1, ET from Asc to valence band of the TpDTz). The effect of Asc in the decay lifetime of the organic material was also assessed in binary mixtures of TpDTz/Asc, showing a decrease of 48%, in the same range observed for the ternary mixture (compare green and red in Figures 7a and 7c, respectively).

**Figure 7:**
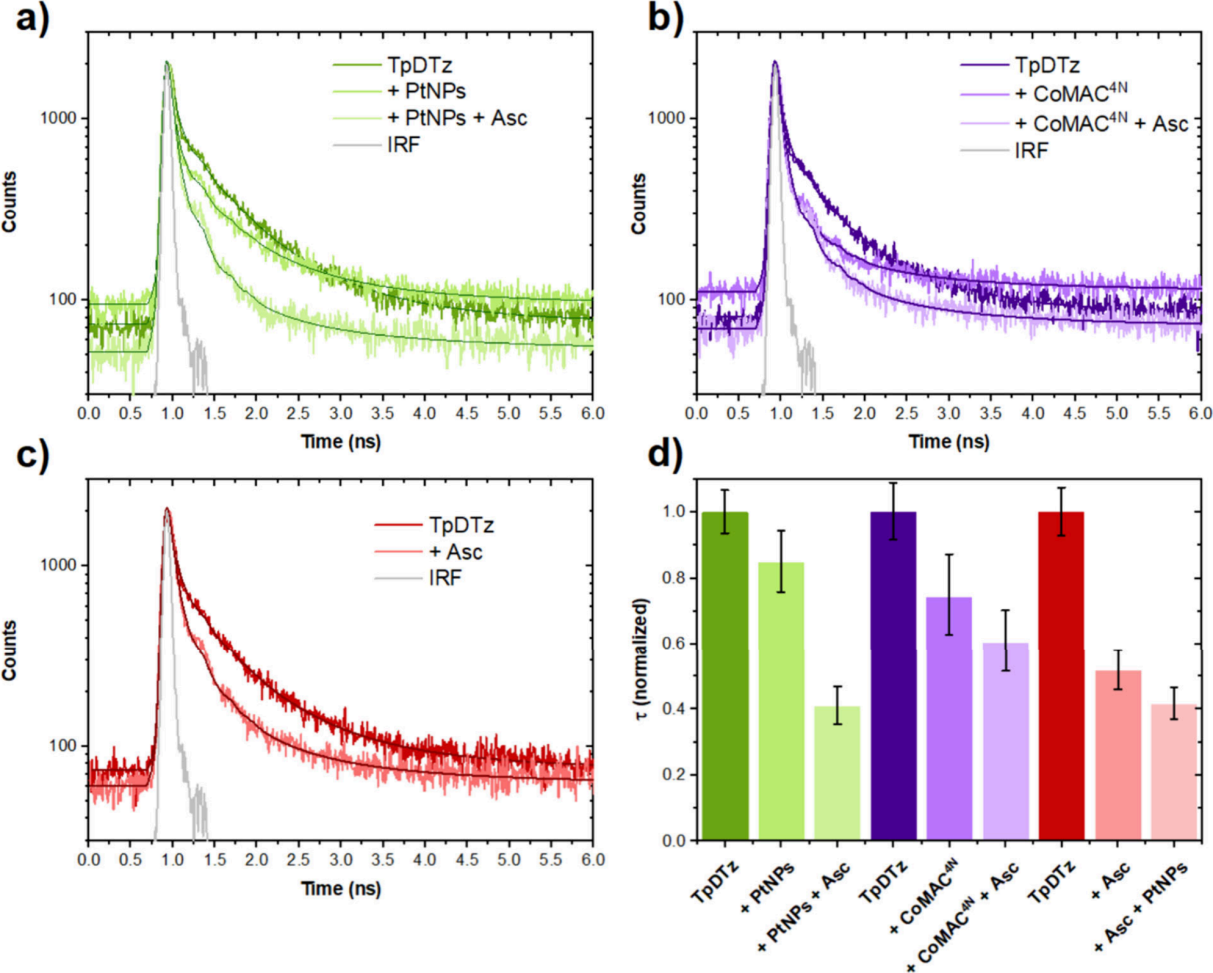
Time correlated single photon counting (TCSPC) experiments of TpDTz (0.5 mg·mL^–1^) in the presence of different catalysts and/or ascorbic acid/sodium ascorbate (Asc) (λ_exc_ = 375 nm; λ_em_ = 620 nm): (a) PtNPs (5% w/w) and Asc (0.03 M); (b) CoMAC^4N^ (1% w/w) and Asc (0.03 M); (c) Asc (0.03 M). (d) Comparison of normalized decay lifetime (τ_av_) extracted from TCSPC experiments shown in (a)–(c) and Figures S17–S19. In green, the system corresponding to TpDTz (0.5 mg·mL^–1^), PtNPs (5% w/w) and Asc (0.03 M), first addition of PtNPs. In purple, the system corresponds to TpDTz (0.5 mg·mL^–1^), CoMAC^4N^ (1% w/w), and Asc (0.03 M). In red, TpDTz (0.5 mg·mL^–1^), Asc (0.03 M) and PtNPs (5% w/w), first addition of Asc.

The fast subnanosecond time scale of these phenomena suggests intraparticle as opposed to interparticle charge transfer processes, the latter relaying on diffusivities of the PtNPs, Asc (or its aggregates) and TpDTz particles.^64,65^ This intraparticle charge transfer implies a high affinity of the catalytic species (PtNPs or CoMAC^4N^) and Asc with the surface of the TpDTz material, presumably favored by the high number of heteroatoms in the 2D-COF structure containing the thiazolo­[5,4-*d*]­thiazole and phloroglucinol moieties. HR-TEM analysis of the material after photocatalysis confirms that the PtNPs are well dispersed through the organic framework after washing steps, supporting the formation of a hybrid TpDTz@PtNPs material responsible for the catalysis (Figures S28 and S30).

A similar scenario is observed for the CoMAC^4N^ system, with a reduction of decay average lifetime of 26% when the catalyst is added and a total reduction of 40% for the full ternary mixture Asc/TpDTz/CoMAC^4N^ (Figure 7b). As discussed above for the PL analysis, two different electron transfer processes to the catalyst are assessed for the binary and ternary systems. In the binary TpDTz/CoMAC^4N^ mixture, the electron transfer from TpDTz* to the cobalt, reducing Co­(III) to Co­(II) is interrogated. In contrast, in the ternary Asc/TpDTz/CoMAC^4N^ mixture, TpDTz* reduces Co­(II) to Co­(I) (Scheme S3). Additionally, in the latter system, the contribution of energy transfer from TpDTz* to the Co­(II) complex should also be considered based on the overlapping between TpDTz emission and reduced CoMAC^4N^ absorption spectra (Figures S8 and S13).

Overall, the results summarized in Figures 6 and 7 highlight the favored and fast interactions between the TpDTz material, catalyst PtNPs or CoMAC^4N^ and the ascorbate buffer. These interactions are key in activating the catalyst responsible for performing the chemical reaction and replenishing the valence band by ascorbate, thus reducing the chances of undesirable back-electron transfer processes.

## 3 Conclusions

TpDTz has been used as a model photoactive material and combined with three types of controlled catalytic species including highly active PtNPs with an average diameter of 2.7 ± 0.4 nm. HER electrocatalytic performance of the PtNPs is demonstrated by cyclic voltammetry analysis, showing excellent results at both pH 4.1 (ascorbate buffer) and pH 10.9 (10% TEOA), as expected for small size nanoparticles based on platinum metal. Surprisingly, these PtNPs are inactive under photocatalytic conditions when combined with TpDTz at pH 10.9 and using TEOA as SED. These results agree with previous results using TpDTz and Pt nanoparticles formed *in situ* from H_2_PtCl_6_ but contrast with many other reported systems that show significant activity using Pt catalysts and TEOA as SED. In sharp contrast, our PtNPs show outstanding HER photocatalytic activity when ascorbate is used as SED at pH 4.1 under solar light irradiation (15 900 μmol H_2_ g^–1^ h^–1^), ruling out a poisoning of the Pt catalyst by TpDTz. Instead, these results have been rationalized by means of PL and TCSPC experiments that prove the efficient quenching of TpDTz fluorescence by the PtNPs and ascorbate SED but poor quenching with the TEOA SED. Finally, the catalytic activity is further enhanced by using a light source centered at the TpDTz maximum absorbance (λ_max_ = 440–550 nm) and far from the absorbance of the PtNPs catalyst (λ < 300 nm), minimizing detrimental pathways and achieving a benchmarking catalytic rate of 106 000 μmol H_2_ g^–1^ h^–1^ using 5% Pt w/w. Analysis of TpDTz material after one hour of photocatalysis confirm that the PtNPs are attached to the organic framework forming the hybrid material TpDTz@PtNPs, which is responsible for the catalysis. The understanding of the Pt based systems allowed us to identify suitable noble metal-free catalysts that resulted in high catalytic activity. Particularly, CoMAC^4N^ achieves an impressive catalytic rate of 10 400 μmol H_2_ g^–1^ h^–1^ under optimized conditions including the use of 1% Co w/w, ascorbate buffer and LED light illumination (λ_em_ = 525 nm). Importantly, this rate is on the same order of magnitude and only three times slower than that obtained with the noble metal based PtNPs system studied here when the same amount of metal is used (1% Pt w/w, 28 300 μmol H_2_ g^–1^ h^–1^). Overall, these results highlight the potential of well-defined catalytic centers for studying charge-transfer events in light-induced hydrogen evolution catalysis in 2D COFs and other organic semiconductors. Such an approach enables precise control over key parameters, leading to enhanced photocatalytic performance.

## 4 Experimental Section

### 4.1 General Considerations

All commercial reactants were used without further purification. Tp (2,4,6-trihydroxybenzene-1,3,5-tricarbaldehyde),^66^ PtNPs,^29^ Co-MAC^4N^ precursor^33,37^ and Co-MAC^5N^ precursor^67^ were prepared following procedures reported in the literature. All anhydrous solvents were dried over 3 Å molecular sieves. The synthesis of TpDTz was performed in a Carousel 12 Plus Reaction Station from Radleys. The specification of the Teflon filter used is as follows: PTFE, pore size 0.45 μm, o̷ = 47 mm. The photocatalytic experiments were run in a quartz or pyrex (depending on the light source) jacketed reactor with a total volume of 10 mL. Pressure (1 atm) and temperature (25 °C) inside the reactor were considered constant during the experiment. The light source consisted of either a Xe lamp (LS 150 from ABET technologies) equipped with a AM 1.5G filter or a light emitting diode (LED, λ_em,max_ = 525 nm, PR160L Kessil), both light sources calibrated to 100 mW·cm^–2^ using a thermal sensor and Si photodiode. Hydrogen gas was measured using either a Clark-type sensor from UNISENSE or Agilent Technologies gas chromatograph (GC) equipped with molecular sieves column and TCD detector. The experimental setups are shown in Figure S20.

### 4.2 Synthesis of COF Precursors

#### 4.2.1 N,N’-(Thiazolo­[5,4-*d*]­thiazole-2,5-diylbis­(4,1-phenylene))­diacetamide (1)

To a round-bottom flask equipped with a magnetic stirring bar were added 4-acetamidobenzaldehyde (1.63 g, 10 mmol, 1.0 equiv) and dithiooxamide (0.601 g, 5 mmol, 1.0 equiv) were added. Subsequently, 20 mL of dry DMF (c_aldehyde_ = 0.5 M) were added. Next, a Dimroth condenser was coupled to the flask, and the reaction was heated to 140 °C for 5 h while stirring. Upon completion, the reaction mixture was allowed to cool down overnight. The flask contents were filtered, and the solid was washed further with cold DMF and finally with abundant Et_2_O. The solid was subjected to high vacuum for complete dryness and was obtained pure without needing a further purification process. The product is obtained as pale-yellow crystals (0.520 g, 1.27 mmol, 25%). ^**1**^ **H NMR** (400 MHz, DMSO-*d*_6_) δ (ppm) = 10.25 (s, 2H), 7.96 (d, *J* = 8.4 Hz, 4H), 7.76 (d, *J* = 8.4 Hz, 4H), 2.09 (s, 6H). ^**13**^ **C {** ^**1**^ **H} NMR** (101 MHz, DMSO-*d*_6_) δ (ppm) = 168.8, 168.1, 141.7, 127.8, 127.0, 119.3, 24.2. **FT-IR** (cm^–1^, neat, ATR), *ṽ* = 3465, 3043, 1658, 1597, 1519, 831, 612. **HRMS (ESI)** calcd for C_20_H_16_N_4_O_2_S_2_Na^+^ [M + Na]^+^: 431.0607, found 431.0621.

#### 4.2.2 4,4’-(Thiazolo­[5,4-*d*]­thiazole-2,5-diyl)­dianiline (DTz)

To a round-bottom flask equipped with a magnetic stirring bar thiazolo­[5,4-*d*]­thiazole acetamide derivative **1** (1.0 g, 2.4 mmol, 1.0 equiv) was added. Subsequently, 12 mL of HCl 37% and 12 mL of glacial acetic acid were added (c = 0.1 M). A Dimroth condenser was coupled to the flask and the reaction was stirred at 120 °C for 72 h. After the reaction completion, the flask was cooled down using an ice–water bath. Subsequently, aqueous ammonia solution (32%) was slowly added until pH = 9 while a steam of N_2_ was introduced in the flask. The content of the flask was then filtered with a Nylon filter and washed with gentle amounts of water and 25 mL of cold EtOH. The filtered solid was further dried under high vacuum, obtaining the final product as a yellow solid (0.75 g, 2.3 mmol, 96%). ^**1**^ **H NMR** (300 MHz, DMSO-*d*_6_) δ (ppm) = 7.65 (d, *J* = 8.7 Hz, 4H), 6.64 (d, *J* = 8.7 Hz, 4H), 5.84 (s, 4H).^24^

### 4.3 Preparation of TpDTz

The reaction was carried out in a multireactor that could run with up to 12 tubes simultaneously. Usually, more than 2 tubes were prepared at the same time and labeled as part of the same batch. In a single multireactor tube, 60 mg (0.186 mmol) of 4,4’-(thiazolo­[5,4-*d*]­thiazole-2,5 diyl)­dianiline (DTz) and 25.8 mg (0.126 mmol) of 2,4,6-trihydroxybenzene-1,3,5-tricarbaldehyde (Tp) were introduced. Then, three pump-fills with Ar were performed and, with positive Ar pressure, 0.3 mL of aqueous acetic acid 6 M and 6 mL of a 1:3 mixture of *o*-dichlorobenzene:*N*,*N*-dimethylacetamide were added. The system was heated at 150 °C for 72 h without stirring. Afterward, the reaction mixture was cooled down to room temperature and the solid formed was filtered under vacuum using a Teflon filter. The collected solid was washed with THF (20 mL × 3), acetone (20 mL × 3) and dried under vacuum, yielding 78 mg of a red powder corresponding to the TpDTz material. **PXRD**, 2θ = 2.71, 4.55, 5.33, 6.91, 8,85, 26.18. **FT-IR** (cm^–1^, neat, ATR), *ṽ* = 3339, 3208, 3018, 1618, 1568, 1440, 1253, 1175, 1093, 1004, 886, 825, 719, 613 cm^–1^. See also additional characterization shown in Figures S1–S4, consistent with reported data.^24^

### 4.4 Preparation of PtNPs

Pt nanoparticles (PtNPs) were prepared following a modified method previously reported.^29^ Briefly, 6 mL of an aqueous solution of potassium hexachloroplatinate (K_2_PtCl_6_, 16 mM) was added to a stirring solution of sodium citrate in water (2.2 mM, 54 mL). After 1 min, 2 mL of a 100 mM freshly prepared ice-cold sodium borohydride aqueous solution was rapidly injected. The solution immediately turned dark brown, indicating the formation of well-dispersed PtNPs. The reaction was stirred continuously for 60 min to ensure completion. HR-TEM analysis shows a monodisperse distribution of PtNPs with average diameter of 2.7 ± 0.4 nm (Figure S5) that were used without further purification (PtNPs concentration: 322 mg/L by ICP-OES analysis).

### 4.5 Light-Induced Hydrogen Evolution Reaction

#### 4.5.1 General Procedure

In a 10 mL jacketed reactor equipped with a magnetic rod, 317 mg (1.6 mmol) of sodium ascorbate and 282 mg (1.6 mmol) of ascorbic acid were added. Then, in a separated vial, a suspension of 1 mg of TpDTz in 1 mL of Milli-Q water was sonicated for 1 min. This suspension was then transferred to the jacketed reactor, and the vial used to make the suspension was rinsed with 1 mL of additional Milli-Q water, which was added to the jacketed reactor. Subsequently, an aqueous solution of catalyst was added to the mixture (161 μL of PtNPs, 322 mg/L), 167 μL of H_2_PtCl_6_ (0.0016 M), 100 μL of CoMAC^4N^ precursor (0.002 M) or 100 μL of CoMAC^5N^ precursor (0.002 M)). The reactor was closed with a septum, and the system was degassed by bubbling N_2_ into the solution for 5 min. The degassed reactor was irradiated and the evolution of hydrogen followed online with a Clark sensor located at the headspace of the reactor or by taking aliquots of the headspace and injecting them into a GC approximately every 20 min intervals.

#### 4.5.2 Recycling Study

In a 100 mL jacketed reactor equipped with a magnetic rod, 6.34 g (32 mmol) of sodium ascorbate and 5.64 g (32 mmol) of ascorbic acid were added. Then, in a separated vial, a suspension of 20 mg of TpDTz in 20 mL of Milli-Q water was sonicated for 1 min. This suspension was then transferred to the jacketed reactor, the vial used to make the suspension was rinsed with 18 mL (when CoMAC^4N^ used) or 16.8 mL (when PtNPs used) of additional Milli-Q water, which was added to the jacketed reactor. Subsequently, an aqueous solution of catalyst was added to the mixture (2 mL of CoMAC^4N^ precursor (0.002 M) or 3.2 mL of PtNPs (317 mg/L). The reactor was closed with a septum, and the system was degassed by bubbling N_2_ into the solution for 15 min. The degassed reactor was irradiated with a LED light (λ_em_= 525 nm), and the evolution of hydrogen followed online with a Clark sensor located at the headspace of the reactor. After one hour, the light is turned off, the supernatant solution is removed by centrifugation. The remaining solid was washed by combining centrifugation and vortex agitation with twice with 10 mL of MeOH and once with 10 mL of DCM. The solid was then dried overnight in a vacuum oven at 60 °C, recovered and reused in the next experiment.

### 4.6 Photoluminescence Quenching and Time Correlated Single Photon Counting Experiments

In a 3 mL quartz cuvette, 2.5 mL of a freshly prepared suspension of TpDTz 0.5 mg/mL were added followed by PL or TCSPC measurement. Sequentially, known amounts of aqueous stock solutions containing the desired component (catalyst or SED) were added to the TpDTz suspension, followed by PL or TCSPC measurement. In the case of SED (Asc and TEOA), 1.6 M stock solutions in Milli-Q water were prepared. Regarding the PtNPs, a 300 mg/L suspension was used. In the case of CoMAC^4N^ and CoMAC^5N^, 0.02 M stock solutions in Milli-Q water were prepared. All the needed amounts of SED and catalyst were added on top of the TpDTz suspension by using a micropipette. For the PL experiments, the resulting mixture was manually mixed and measured directly. In the case of the TCSPC experiments, the mixture was magnetically stirred throughout the whole experiment. Photoluminescence emission spectra and lifetimes were recorded using a double monochromator spectrofluorometer (Edinburgh FLS980, see SI for detailed information). Steady-state measurements were performed with an excitation wavelength of 500 nm using an appropriate long-pass filter. For time-resolved measurements, the excitation wavelength was set to 375 nm, and the emission was collected over a bandwidth of 0.08 nm centered at the emission peak. The decay curves were fitted using a model combining a delta function and a gamma function.^68^

## Supplementary Material



## Notes

Additional experimental procedures and equipment details. Spectroscopic, crystallographic, morphological, porosity, electrochemical, TCSPC data fitting, and additional photocatalytic data (PDF)
