# Supplementary material for: The Power of Catalytic Centers and Ascorbate in Boosting the Photocatalytic Hydrogen Evolution Performance of TpDTz 2D-COF
Source: J Am Chem Soc. 2025 Dec 22;148(1):1316–28. doi: 10.1021/jacs.5c17806 (PMC12814356; doi:10.1021/jacs.5c17806)
Supplement: Supplementary file 1 [file ja5c17806_si_001.pdf]

The Power of Catalytic Centers and Ascorbate in Boosting the Photocatalytic Hydrogen Evolution Performance of TpDTz 2D-COF

David Reyes-Mesa,[a] Pau Sarró,[a] Muriel F. Gusta,[b] Alberto Jiménez-Solano,[c] Saunak Das,[d] Bishnu P. Biswal,[d,e,f] Hugo A. Vignolo-González,[d] Laura Velasco-Garcia,[a] Antoni Llobet,[g] Neus G. Bastús,[b] Víctor Puntes,[b] Adelina Vallribera,[a] Roser Pleixats,[a] Albert Granados,[a] Bettina V. Lotsch,[d,h,i,] Carolina Gimbert-Suriñach[a,]**

[a]Department of Chemistry and Centro de Innovación en Química Avanzada (ORFEO-CINQA), Universitat Autònoma de Barcelona, Cerdanyola del Vallès, Barcelona 08193, Spain.

[b]Catalan Institute of Nanoscience & Nanotechnology - ICN2 (BIST and CSIC), Campus UAB, 08193 Bellaterra (Barcelona), Spain.

[c]Departamento de Física, Universidad de Córdoba, Edificio Einstein (C2), Campus de Rabanales, 14071 Córdoba, Spain.

[d]Nanochemistry Department, Max Planck Institute for Solid State Research, 70569 Stuttgart, Germany.

[e]School of Chemical Sciences, National Institute of Science Education and Research (NISER), Khurda 752050 Odisha, India.

[f]Homi Bhaba National Institute (HBNI), Training School Complex, Mumbai 400094, India.

[g] Institute of Chemical Research of Catalonia (ICIQ), Avda. Països Catalans 16, 43007 Tarragona, Spain.

[h] Department of Chemistry, University of Stuttgart, 70569 Stuttgart, Germany.

[i] Department of Chemistry, University of Munich (LMU), 81377 Munich, Germany.

Table of Contents

		Page
**1**	General considerations	S3
**2**	Synthesis of DTz and TpDTz	S4
**3**	PXRD, HR-TEM, BET and IR characterization of TpDTz 2D-COF	S5
**4**	PtNPs HR-TEM	S7
**5**	UV-Vis and diffuse reflectance spectroscopy	S8
**6**	Electrochemical analysis of catalytic species	S10
**7**	Photoluminescence (PL) and Time Correlated Single Photon Counting (TCSPC)	S13
**8**	Scheme of the CoMAC^{4N} catalyzed HER reaction	S18
**9**	Photocatalysis experiment set-up	S19
**10**	Additional HER photocatalytic data	S20
**11**	Post-catalysis characterization	S25
**12**	Estimation of VB and CB of TpDTz	S29
**13**	Comparative of relevant HER photocatalytic systems	S31
**14**	NMR spectra	S34
**15**	References	S36

1. General Considerations

All **Nuclear Magnetic Resonance (NMR)** spectra of the synthesized molecules have been measured in *Servei de Ressonància Magnètica Nuclear* of *Universitat Autònoma de Barcelona*. ^{1}H-NMR and ^{13}C-NMR have been obtained using the instruments *Bruker Ascend 300/400/500 MHz - NEO* or *Bruker 400/600 MHz - Avance III*. Chemical shifts (δ) have been expressed in ppm and the coupling constants (*J*) in Hz. Residual solvent (($(CD_3)_2SO$, δ_H, 2.50 and δ_C, 39.52 ppm and $CDCl_3$, δ_H, 7.26 and δ_C, 77.16 ppm) have been used as an internal reference to calibrate the ^{1}H-NMR or ^{13}C-NMR spectra. **The Inductively Coupled Plasma – Optical Emission Spectrometry (ICP-OES)** analysis were measured on an Agilent 5900 spectrometer by the *Servei d'Anàlisi Química* at the *Universitat Autònoma de Barcelona*. The **Infrared spectra (IR)** were measured either using the spectrometer *Bruker Alpha II* with the accessory *Platinum Gate ATR*. The **Powder X-ray diffraction (PXRD)** patterns were measured at room temperature on a PANalytical® X'Pert PRO diffractometer by the *Servei de Difracció de Raigs X* at the *Universitat Autònoma de Barcelona*. Whenever it is indicated in the procedure, the experiments were performed under standard vacuum procedures using Schlenk lines under Argon or Nitrogen atmosphere, using anhydrous and degassed solvents by doing the pump-fill methodology and transferred with syringes. The **cyclic voltammetry (CVs)** experiments were performed with a BioLogic® SP-50 Single Channel Potentiostat, in one-compartment three-electrode set-up using glassy carbon disk as working electrode (ø = 3 mm), platinum wire as auxiliary electrode and SCE as reference electrode. Experiments were performed at room temperature, using water as solvent, degassing with Ar, using Na_2SO_4 as supporting electrolyte (0.1 M). The potentials (*E*) in the manuscript are reported vs the Normal Hydrogen Electrode (NHE) calculated according to $E_{NHE} = E_{SCE} + 0.245$ V. The **HR-TEM** analysis was performed in *Servei de Microscòpia de la Universitat Autònoma de Barcelona* with the equipment TEM Jeol 2011. **HR(S)TEM and EDX analysis** was performed at ICN2 Electron Microscopy Unit using a FEI Tecnai F20 S/TEM operating at 200 kV equipped with an energy-dispersive X-ray spectroscopy (EDX) detector system. Samples were prepared by drop-casting a dispersion of PtNPs or COF onto carbon-coated Cu grids, followed by drying for at least 24 h. The **BET** analysis was performed in the *Instituto de Ciencia de Materiales de Barcelona* using ASAP 2020 V4.04. The **Diffuse reflectance UV-Vis** spectra were recorded using a Cary 5000 UV-Vis-NIR Spectrophotometer form agilent (referenced to barium sulphate). **PL** and **TCSPC** experiments were measured with an Edinburgh FLS 980 spectrometer. For steady state emission, samples were excited using a 450 W Xe lamp passed through a single grating (1800 l/mm, 250 nm blaze) Czerny-Turner monochromator and finally a bandwidth slit. Emission from the sample was passed through a double grating (1200 l/mm, 500 nm blaze) Czerny-Turner monochromator and finally detected by a cooled microchannel plate photomultiplier tube (MCP-PMT) detector. Filters of the appropriate wavelength were always used in the excitation and emission channel. The dynamics of emission decay were monitored with the FLS980's time-correlated single-photon counting capability (1024 channels, 10 ns window) with data collection for 2000 counts. Samples were excited with an Edinburgh EPL-375 picosecond pulsed laser diode (375 ± 6 nm, pulse width – 68 ps) and a cooled microchannel plate photomultiplier tube (MCP-PMT) detector. 390 nm filter was always used in the emission channel. TCSPC data were fitted with a combination of an exponential and a distribution function.

2. Synthesis of DTz and TpDTz

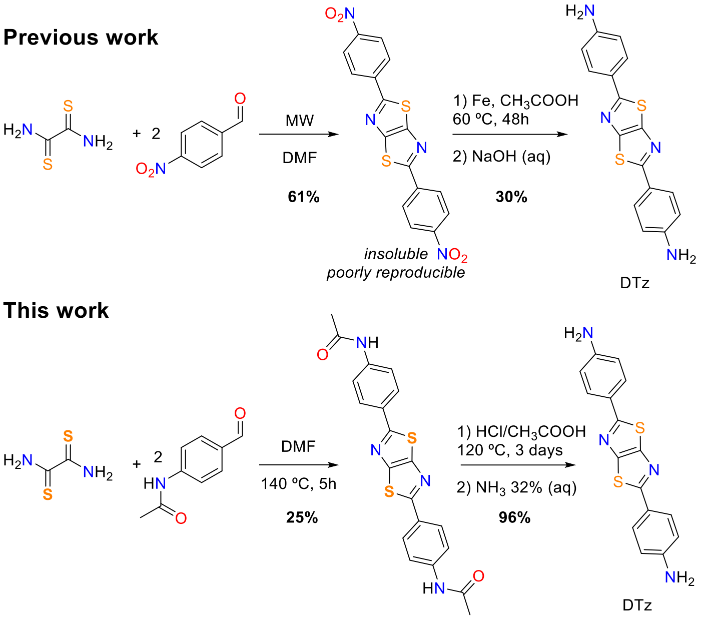

Scheme S1. Synthesis of DTz. **Top)** Previous reported methodology requires the use of very insoluble dinitroderivative.[1] **Bottom)** This work synthesis.

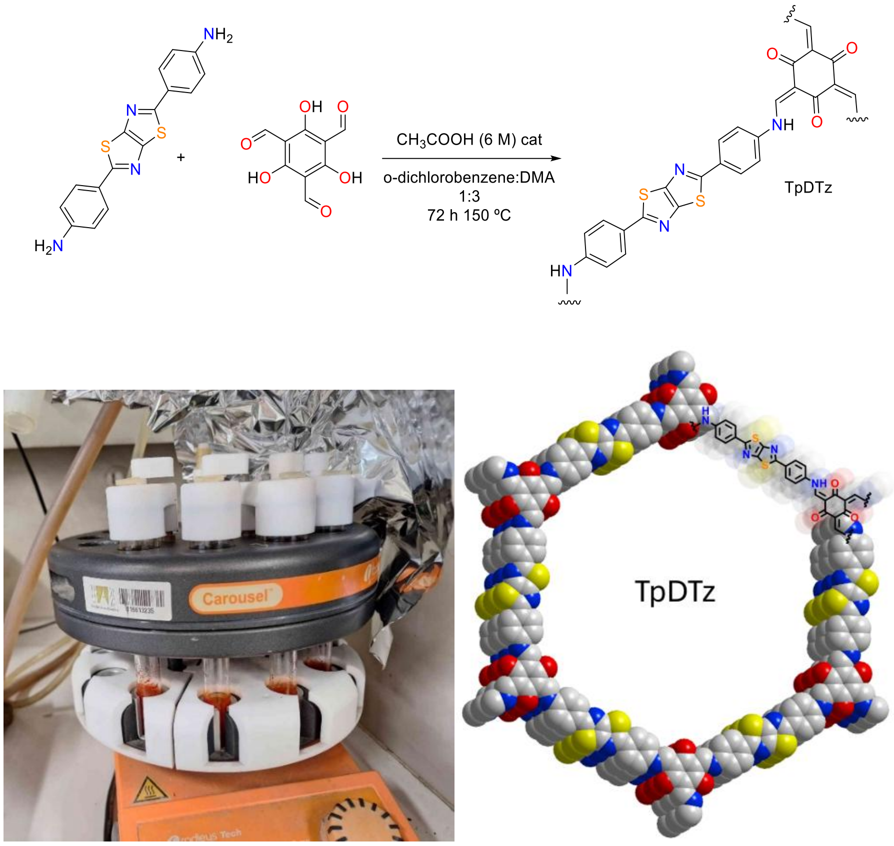

Scheme S2. **Top)** Synthesis of TpDTz 2D-COF. **Bottom)** Multireactor used to perform large scale synthesis (left) and hexagonal structure of TpDTz 2D-COF (right).

3. PXRD, HR-TEM, BET and IR characterization of TpDTz

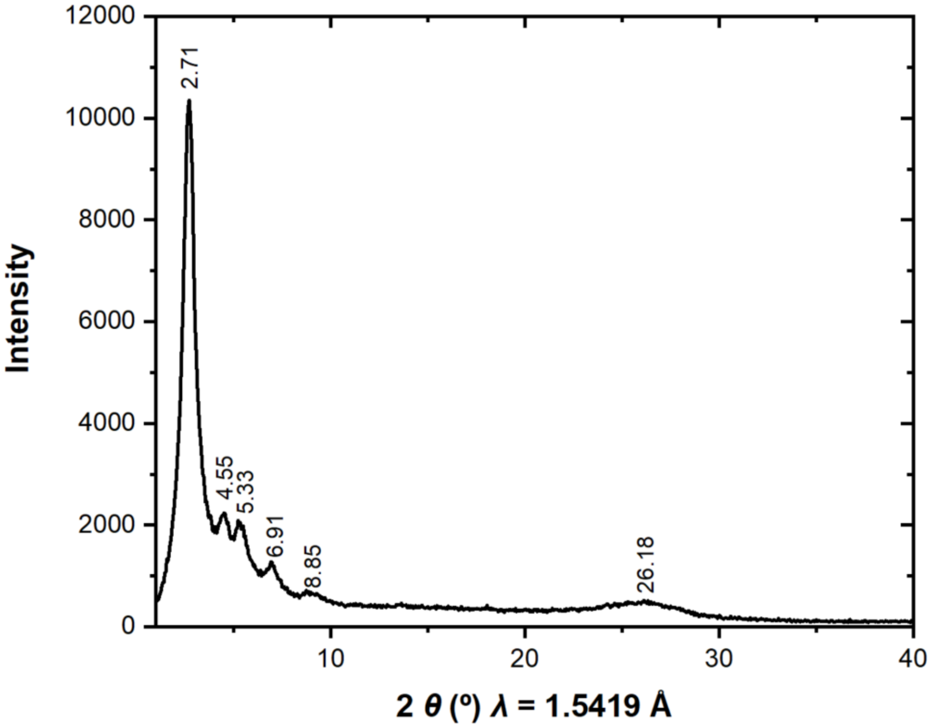

Figure S1. Powder X-Ray Diffractogram of the TpDTz 2D-COF showing the same characteristic pattern as in previous report.[1]

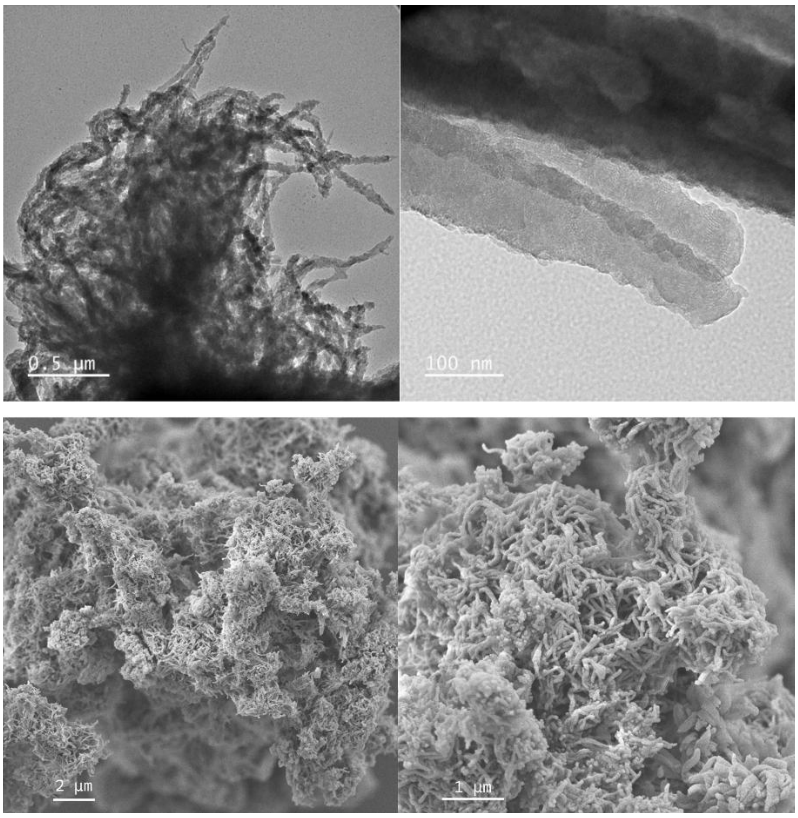

Figure S2. HR-TEM (**top**) and SEM (**bottom**) of TpDTz 2D-COF.

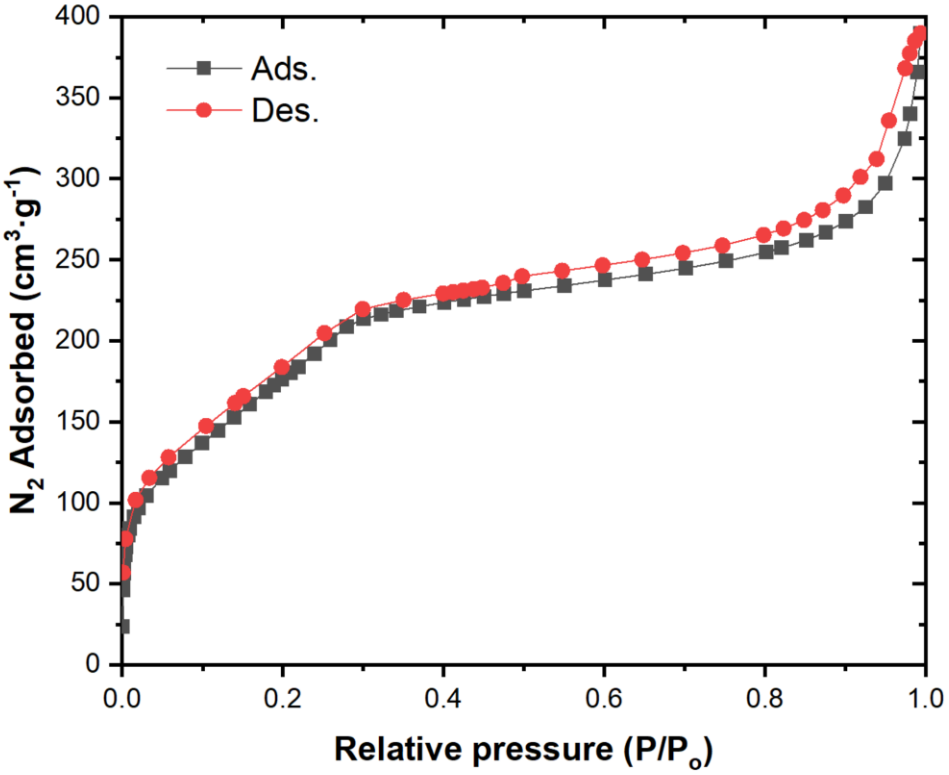

Figure S3. N_2 adsorption-desorption isotherm of TpDTz 2D-COF (BET surface area = 721 ± 10 $m^2 \cdot g^{-1}$).

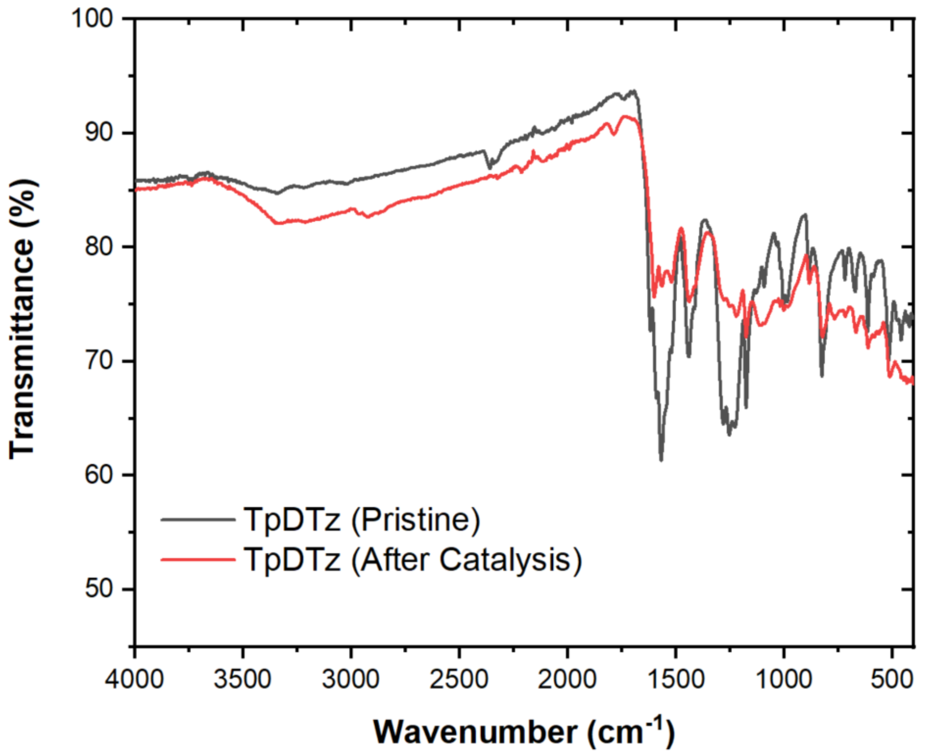

Figure S4. ATR-IR spectrum of TpDTz 2D-COF. Black: pristine material. Red: after 2h of photocatalysis using PtNPs (5 %).

4. PtNPs HR-TEM

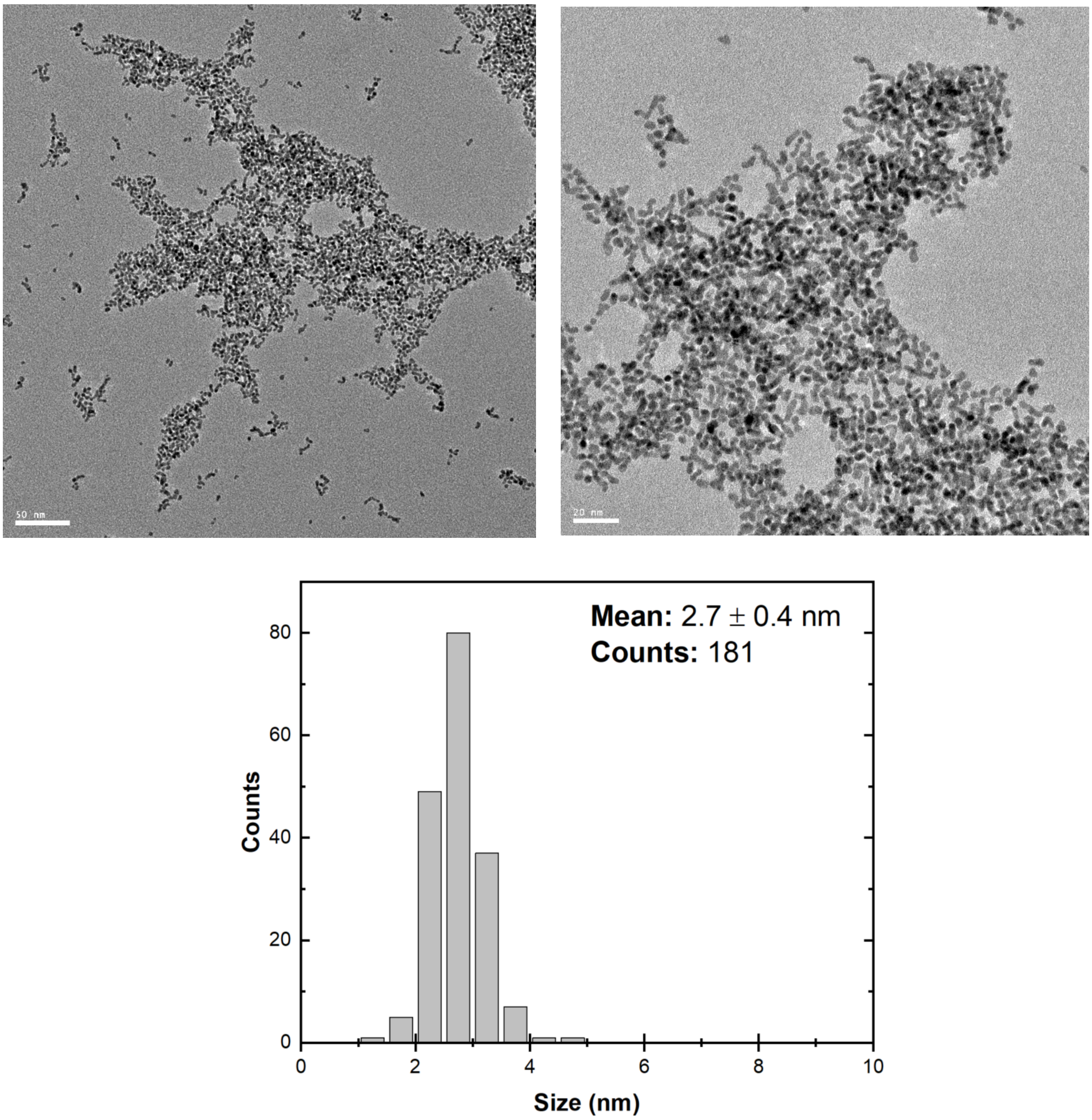

Figure S5. **Top)** HR-TEM images of pre-synthesized PtNPs. **Bottom)** Size distribution analysis.

5. UV-Vis and diffuse reflectance spectroscopy

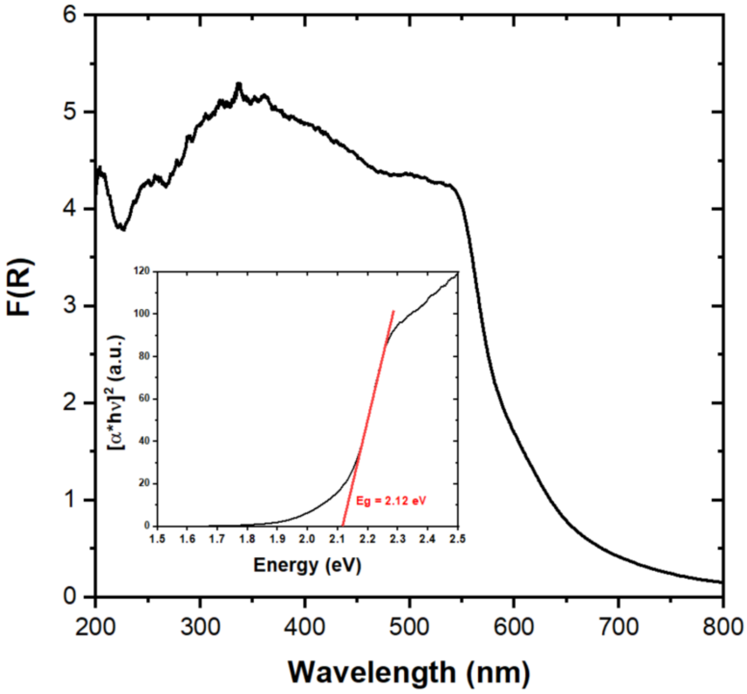

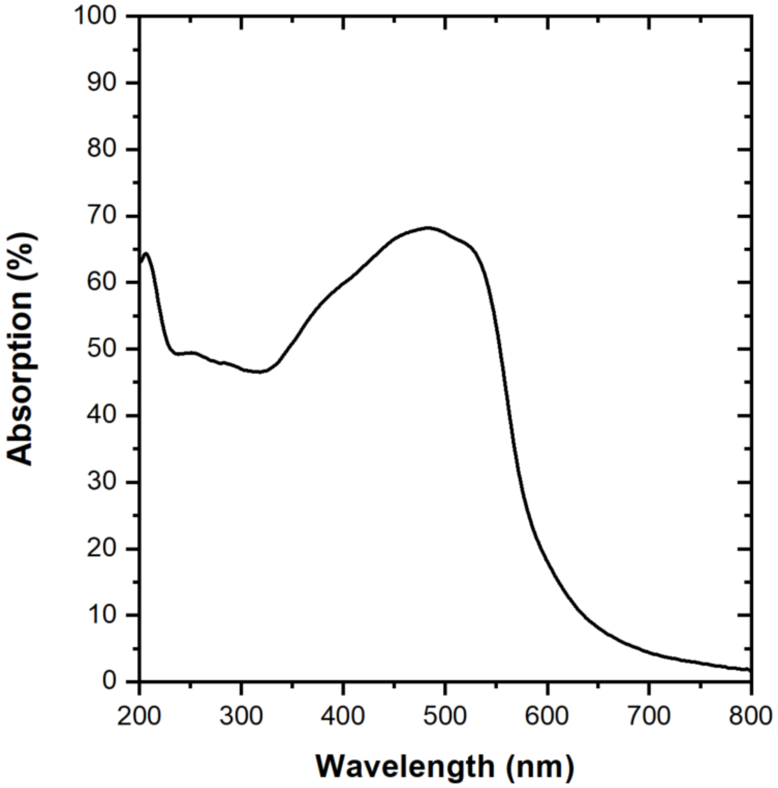

Figure S6. **Top)** DR UV-Vis spectrum of TpDTz 2D-COF (solid state), the inset plot indicates the Kubelka-Munk function for a direct gap, from which an optical band gap of 2.12 eV is extracted. **Bottom)** DR UV-Vis spectrum of a colloidal aqueous solution of 0.5 mg TpDTz 2D-COF · mL^{-1}.

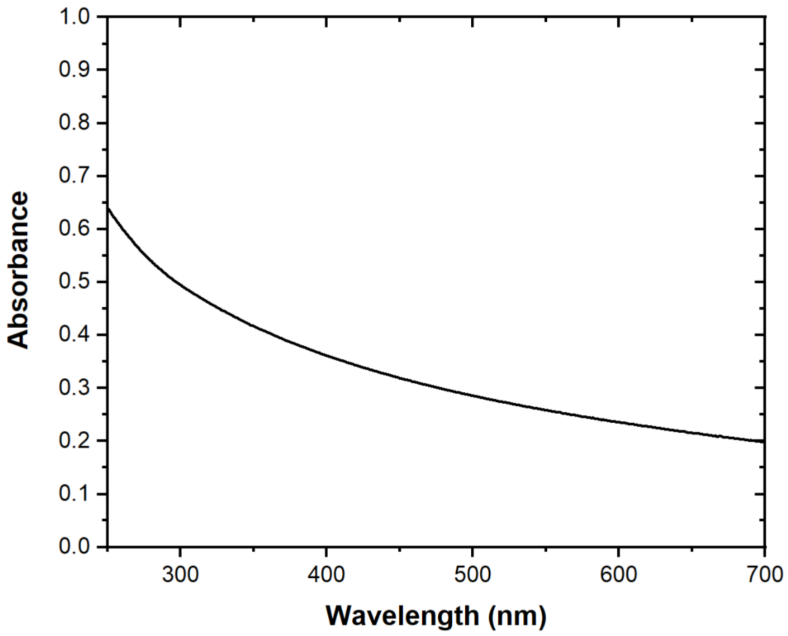

Figure S7. UV-Vis spectrum of PtNPs (0.025 mg · mL^{-1}) in aqueous solution.

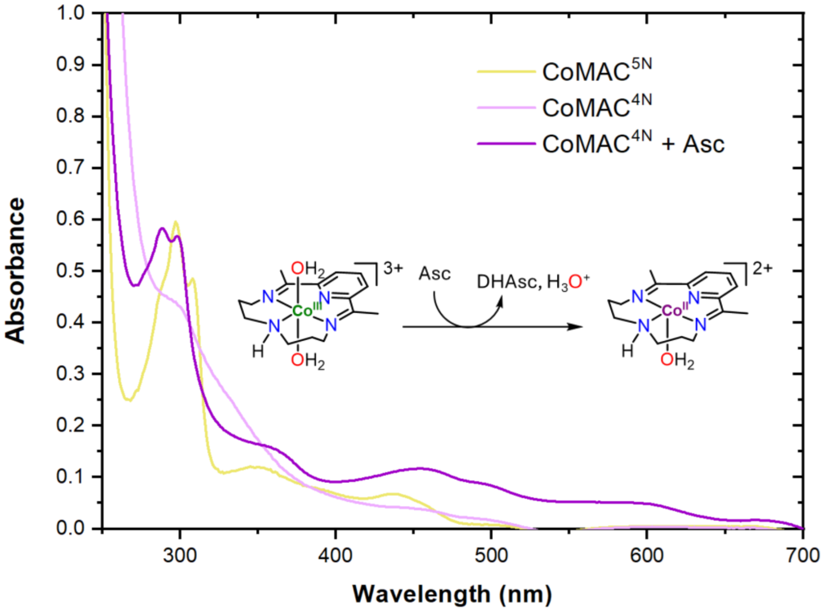

Figure S8. UV-Vis of the cobalt complexes (0.15 mM) in aqueous solution. In yellow $CoMAC^{5N}$. In light purple, $CoMAC^{4N}$. In dark purple $CoMAC^{4N}$ with a 0.1 M ascorbate buffer solution (1:1 mixture of ascorbic acid:sodium ascorbate, Asc). When $CoMAC^{4N}$ is mixed with Asc, the cobalt center in oxidation state Co^{III} is reduced to Co^{II} as illustrated in the *inset* reaction. The new complex shows an expanded absorption up to 700 nm (dark purple trace).[2-4] Note that the labile chlorido ligands exchange by aquo ligands when $CoMAC^{4N}$ is dissolved in water.[3,5] DHAsc is dehydroascorbate, the product of ascorbate oxidation.

6. Electrochemical analysis of catalytic species

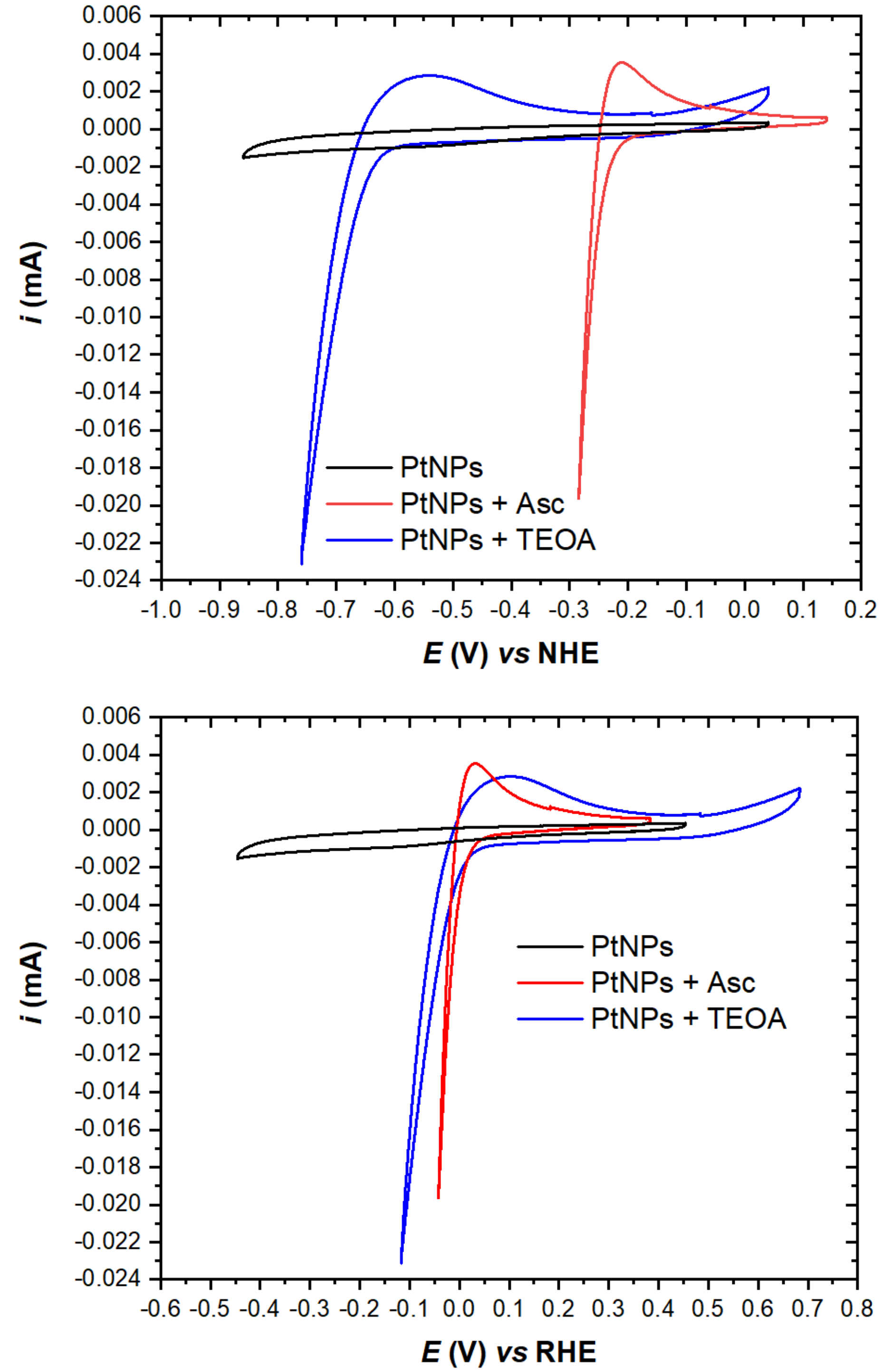

Figure S9. Cyclic voltammograms of a solution of PtNPs (25 mg · L^{-1}) in water-Na_2SO_4 (0.1 M), 100 mV s^{-1} in the absence (black trace) and in the presence of 0.1 M of Ascorbic acid/sodium ascorbate (Asc) (red trace, pH = 4.1) or in the presence of TEOA (blue trace, 10 % (v/v) in water, pH = 10.9). **Top)** NHE scale. **Bottom)** RHE scale.

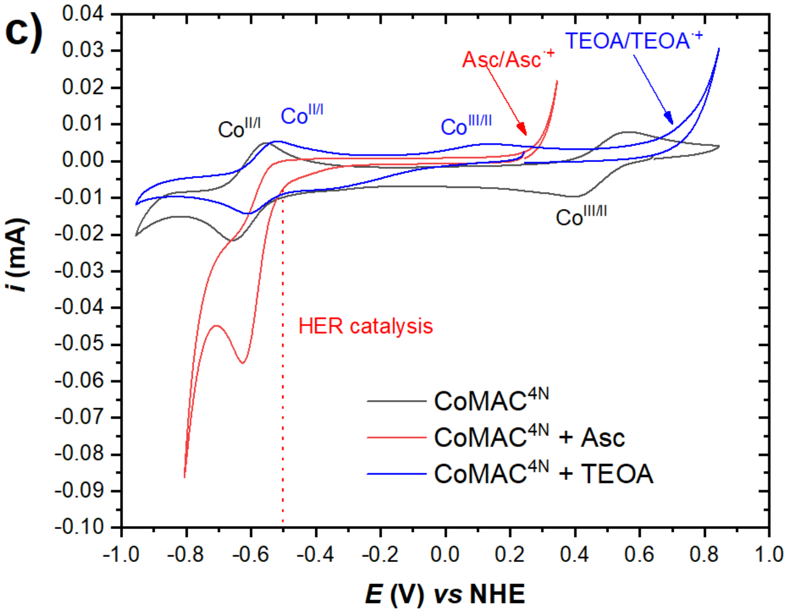

Figure S10. Cyclic voltammograms of CoMAC^{4N} (0.1 mM) in water-Na_2SO_4 (0.1 M), 100 mV s^{-1} in the absence (black trace) and in the presence of 0.1 M of Ascorbic acid/sodium ascorbate (Asc) (red trace, pH = 4.1) or in the presence of TEOA (blue trace, 10 % (v/v) in water, pH = 10.9). When CoMAC^{4N} is mixed with Asc, the cobalt center in oxidation state Co^{III} is reduced to Co^{II}, and the Co$^{III/II}$ redox event is not visible anymore (see reaction *inset* in Figure S8).[2-4]

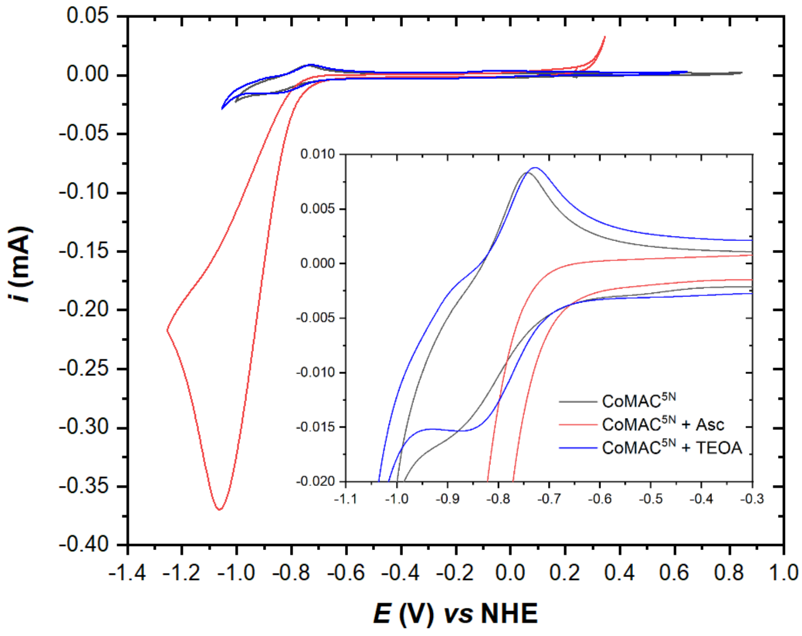

Figure S11. Cyclic voltammograms of CoMAC^{5N} (0.1 mM) in water-Na_2SO_4 (0.1 M), 100 mV s^{-1} in the absence (black trace) and in the presence of 0.1 M of Ascorbic acid/sodium ascorbate (Asc) (red trace, pH 4.1) or the presence of TEOA (blue trace, 10 % (v/v) in water, pH = 10.9).

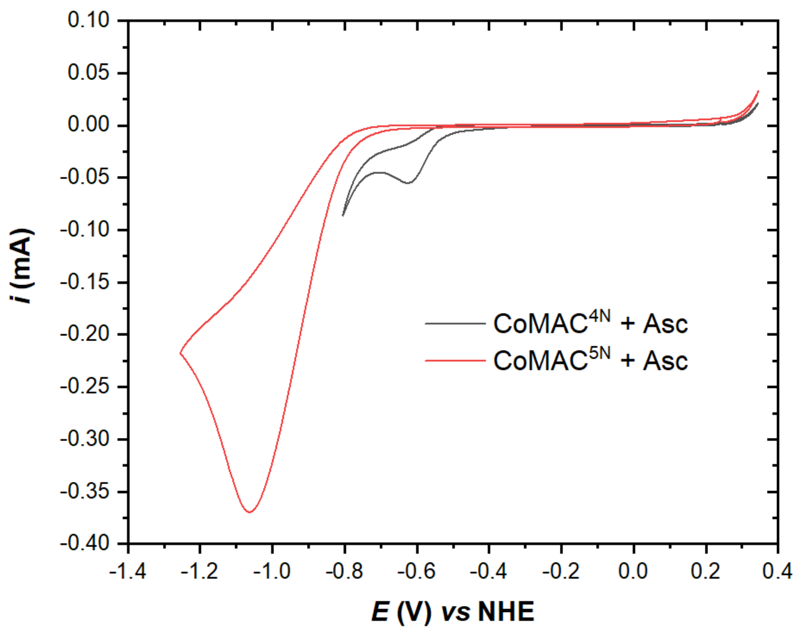

Figure S12. Cyclic voltammograms of 0.1 mM CoMAC^{5N} (red trace) and 0.1 mM CoMAC^{4N} (black trace) in water-Na_2SO_4 (0.1 M), 100 mV s^{-1} in the presence of 0.1 M of Ascorbic acid/sodium ascorbate (Asc, pH 0 4.1).

7. Photoluminiscence (PL) and Time Correlated Single Photon Counting (TCSPC)

Experimental procedure: In a 3 mL quartz cuvette 2.5 mL of a freshly prepared suspension of TpDTz (0.5 mg · mL^{-1}) were added and the PL or TCSPC experiment measured. Sequentially, known amounts of stock solutions of the desired component (catalytic species or sacrificial electron donor) were added. In the case of Asc, a 1.6 M stock solution was prepared by dissolving ascorbic acid (0.704 g, 4.0 mmol) and sodium ascorbate (0.792 g, 4.0 mmol) in 5 mL of MilliQ water. For TEOA, 5 mL of a 1.6 M stock solution were prepared by mixing TEOA (1.056 mL, 8.0 mmol) with MilliQ water (3.944 mL). Regarding the PtNPs, a 322 mg · L^{-1} suspension was used directly as prepared. In the case of $CoMAC^{4N}$ a 0.02 M stock solution was prepared by mixing it (7.8 mg, 0.02 mmol) in 1 mL of MilliQ water and for $CoMAC^{5N}$, it was prepared by mixing $CoMAC^{5N}$ (9.8 mg, 0.02 mmol) in 1 mL of MilliQ water. All the needed amounts of SED and catalytic species were added on top of the TpDTz suspension by using a micropipette. For the PL experiments, the resulting mixture was manually mixed and measured directly. In the case of the TCSPC experiments, the mixture was magnetically stirred along the whole experiment.

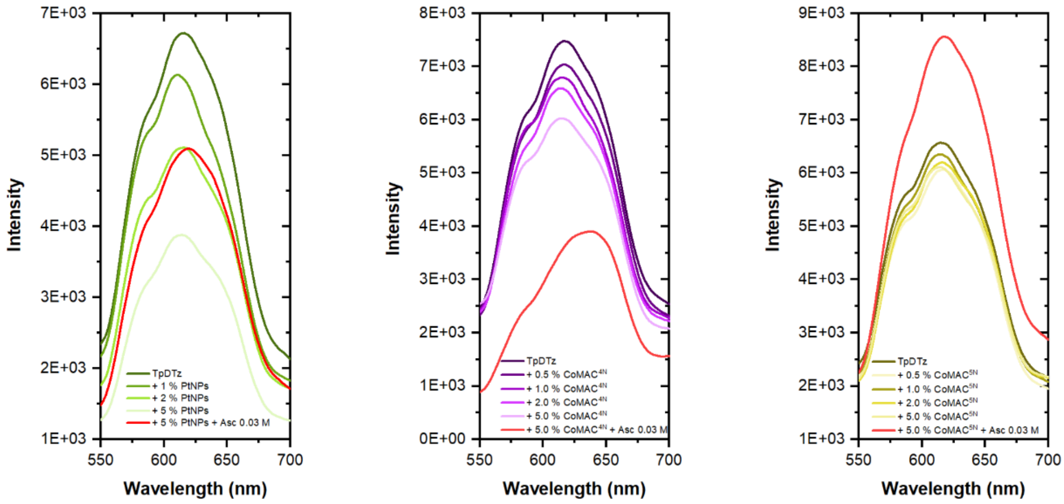

Figure S13. Photoluminescence (PL, λ_{exc} = 500 nm) quenching experiments of TpDTz in water (0.5 mg TpDTz · mL^{-1}) in the presence of different quenchers. **Left**) PtNPs (1-5 %) in green and PtNPs 5 % + Asc 0.03 M in red. **Middle**) $CoMAC^{4N}$ (0.5-5 %) in purple and $CoMAC^{4N}$ 5% + Asc 0.03 M in red. Note that in the binary mixture TpDTz/$CoMAC^{4N}$ we are assessing Co(III) to Co(II) conversion while in the ternary mixture TpDTz/$CoMAC^{4N}$/Asc we are assessing Co(II) to Co(I) and plausible energy transfer (see also Figure S8 and Scheme S3). **Right**) $CoMAC^{5N}$ (0.5-5 %) in yellow and $CoMAC^{5N}$ 5% + Asc 0.03 M in red.

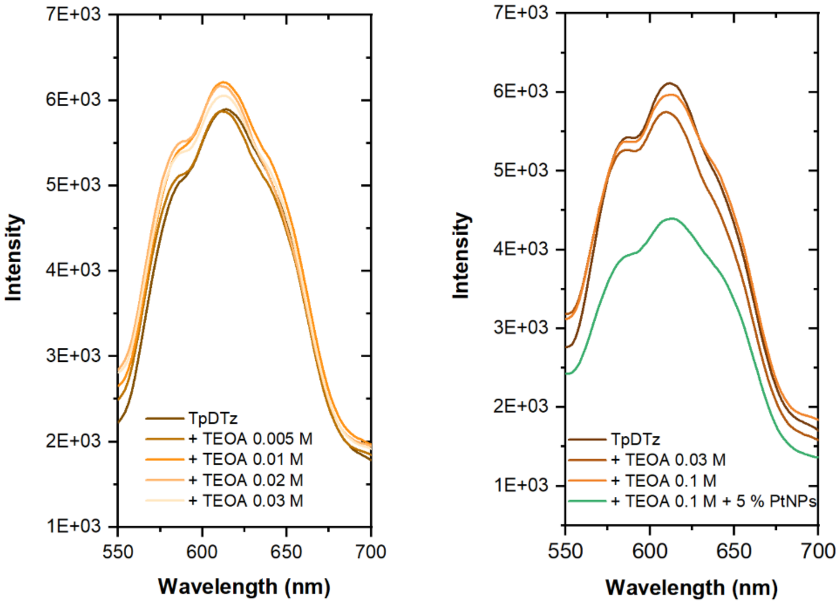

Figure S14. Photoluminescence quenching experiments (PL) of TpDTz in water (0.5 mg TpDTz · mL^{-1}) in the presence of TEOA at different concentrations (λ_{exc} = 500 nm), in the absence (**left**) and in the presence of PtNPs (**right**).

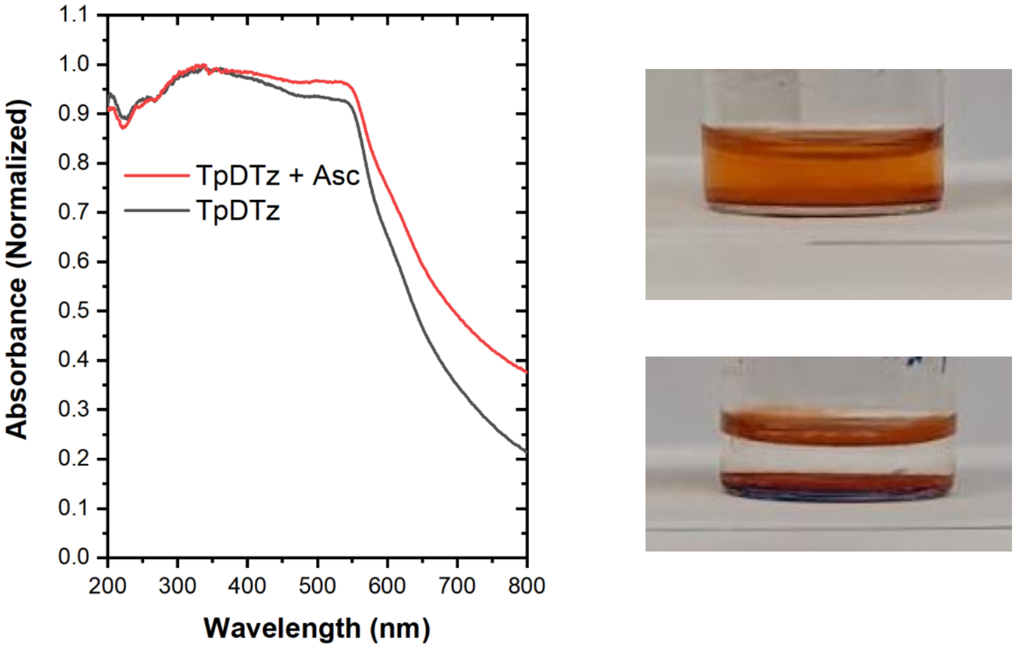

Figure S15. Left) UV-Vis DR spectrum of TpDTz 2D-COF (solid state) before and after adding Asc. **Right)** Sonicated suspension of TpDTz (0.5 mg TpDTz · mL^{-1}) without (top) and with (bottom) addition of Asc.

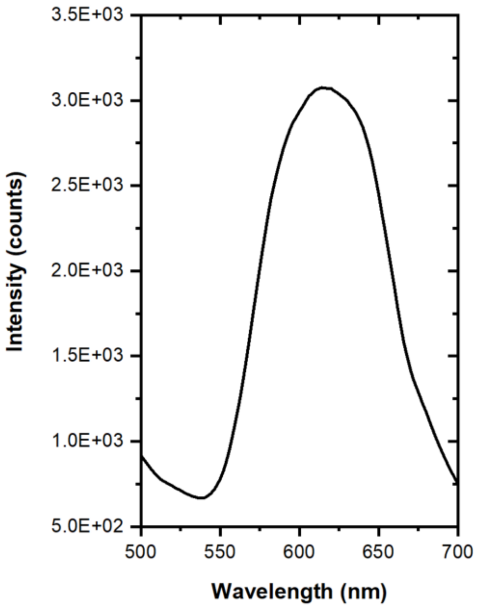

Figure S16. Photoluminescence emission spectrum of TpDTz in water (0.5 mg TpDTz · mL^{-1}) under λ_{exc} = 365 nm.

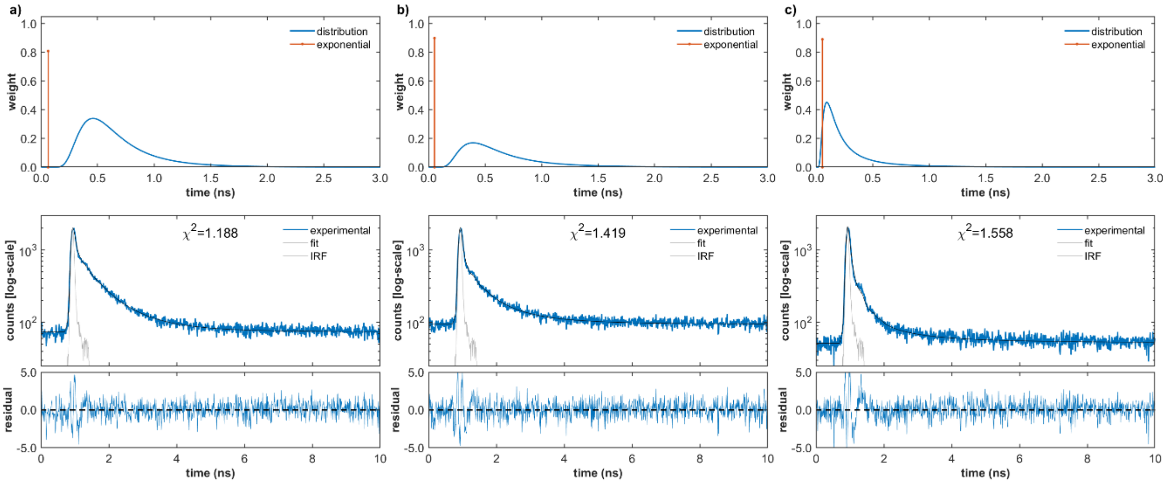

Figure S17. Time correlated single photon counting (TCSPC) experiments of TpDTz (0.5 mg TpDTz · mL^{-1}) in the absence of any quencher (**a**), in the presence of PtNPs (5 % w/w) (**b**) and, in the presence of PtNPs (5 % w/w) and Asc (0.03M) (**c**). First addition of PtNPs and second addition of Asc.

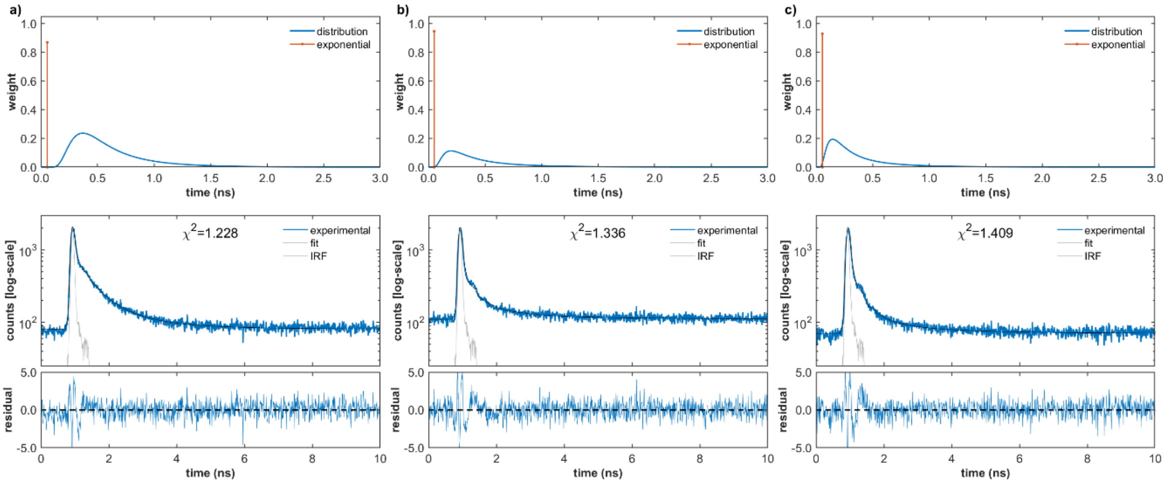

Figure S18 Time correlated single photon counting (TCSPC) experiments of TpDTz (0.5 mg TpDTz · mL^{-1}) in the absence of any quencher (**a**), in the presence of CoMAC^{4N} (1 % w/w) (**b**) and, in the presence of CoMAC^{4N} (1 % w/w) and Asc (0.03M) (**c**).

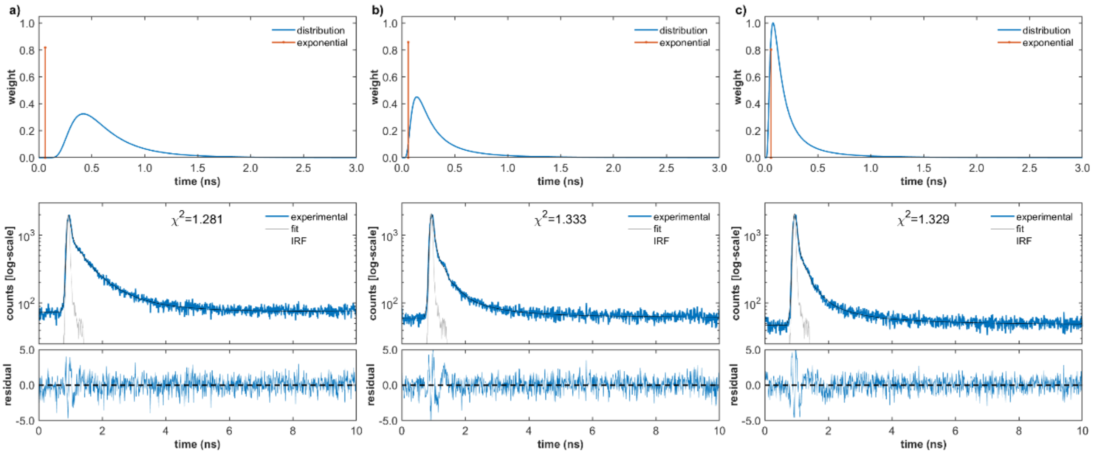

Figure S19. Time correlated single photon counting (TCSPC) experiments of TpDTz (0.5 mg TpDTz · mL^{-1}) in the absence of any quencher (**a**) and, in the presence of Asc (0.03M) (**b**) and, in the presence of PtNPs (5 % w/w) (**c**). First addition of Asc and second addition of PtNPs.

Table S1. TCSPC data fitting.

experiment	exponential		distribution			average	
	weight	τ_{exp} (ns)	weight	τ_{mean} (ns)	τ_{mode} (ns)	τ_{av} (ns)	normalized
TpDTz	0.809	0.063	0.191	0.680	0.460	0.506	1.000
TpDTz + PtNPs	0.900	0.052	0.100	0.686	0.393	0.429	0.847
TpDTz + PtNPs +Asc	0.891	0.056	0.109	0.385	0.095	0.206	0.407
TpDTz	0.869	0.055	0.131	0.641	0.370	0.427	1.000
TpDTz + CoMAC^{4N}	0.947	0.050	0.053	0.669	0.199	0.317	0.742
TpDTz + CoMAC^{4N} + Asc	0.930	0.055	0.070	0.536	0.147	0.258	0.603
TpDTz	0.819	0.061	0.181	0.659	0.421	0.482	1.000
TpDTz + Asc	0.859	0.061	0.141	0.417	0.142	0.249	0.516
TpDTz + Asc + PtNPs	0.803	0.058	0.197	0.308	0.078	0.199	0.413

8. Scheme of the CoMAC^{4N} catalyzed HER reaction

The mechanism of the hydrogen evolution reaction at the cobalt center for the CoMAC^{4N} catalyst has been extensively studied under photocatalytic reactions.[3,4] Based on previous studies and the results obtained in this work, a plausible mechanism for the light-induced hydrogen evolution reaction of the Asc/TpDTz/CoMAC^{4N} system is illustrated in Scheme S3 below, where the cobalt oxidation state is indicated in red below the different species involved in the catalytic cycle. Note that the labile chlorido ligands exchange by aquo ligands when CoMAC^{4N} is dissolved in water.[3,5]

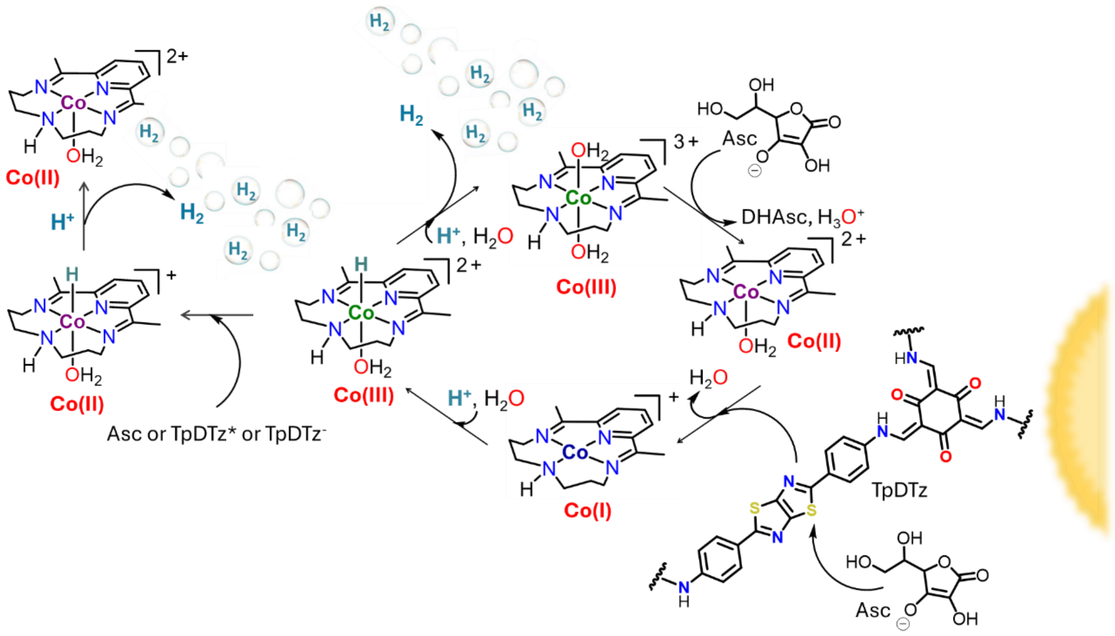

Scheme S3. Proposed light-induced HER steps for the Asc/TpDTz/CoMAC^{4N} system. DHAsc is dehydroascorbate, the product of ascorbate oxidation. The cobalt oxidation state is indicated in red below the different species involved in the catalytic cycle. Note that the labile chlorido ligands exchange by aquo ligands when CoMAC^{4N} is dissolved in water.[3,5]

9. Photocatalysis experiment set-up

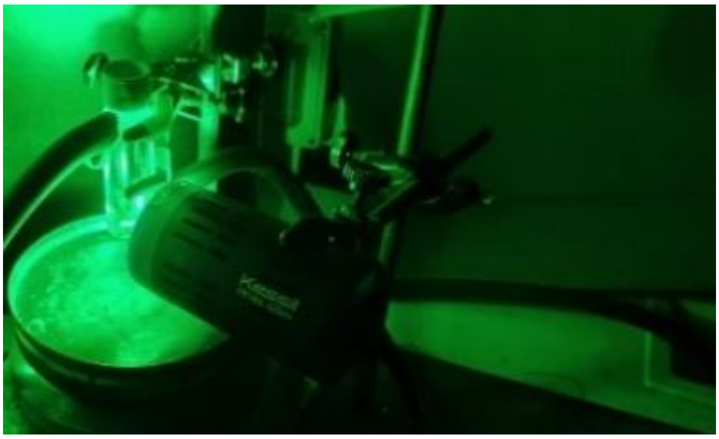

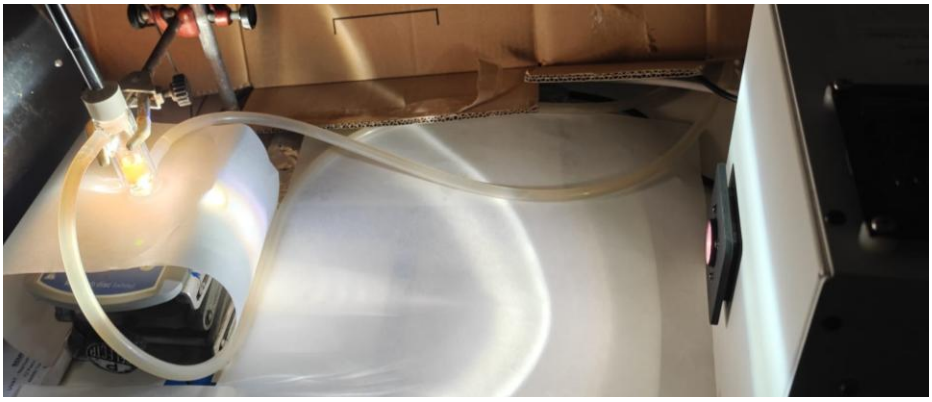

Figure S20. Experimental set-up of the photocatalytic experiments. **Top)** LED $\lambda_{em,max}$ = 525 nm, calibrated at 100 mW · cm^{-2}. **Bottom)** 150 W Xe lamp equipped with AM1.5G filter and calibrated at 100 mW · cm^{-2}.

10. Additional HER photocatalytic data

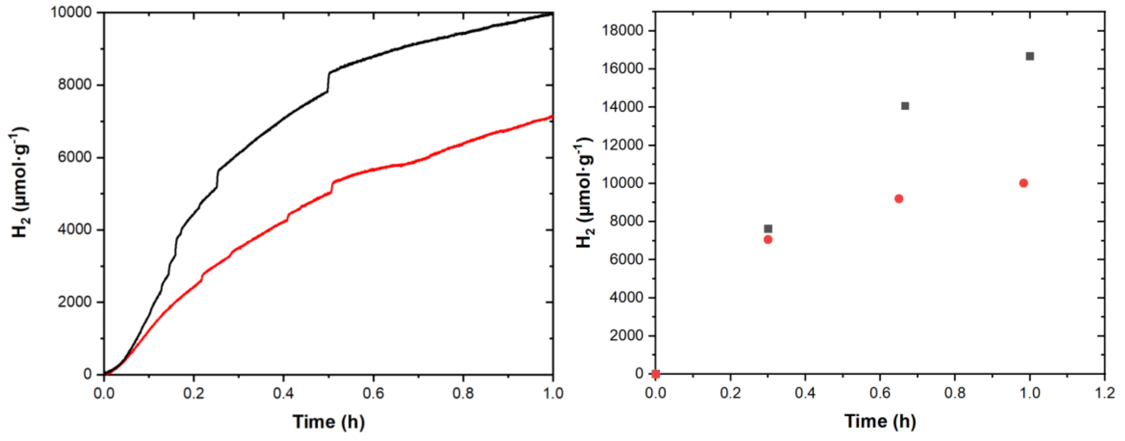

Figure S21. Hydrogen evolution profile of a photocatalytic mixture composed of TpDTz (0.5 mg · mL^{-1}) in a mixture of ascorbic acid/sodium ascorbate buffer 1.6 M and 5 % (w/w) loading of H_2PtCl_6 and irradiated with a 150 W Xe lamp equipped with AM 1.5G filter (100mW · cm^{-2}). **Left)** Two replicates (black and red) where the reaction was followed by Clark sensor. Note that the steps observed in these analyses are due to fast bubble formation that leads to the sudden signal increase when the bubble reaches the sensor located at the headspace of the reactor, see Figure S20. **Right)** Two replicates (black and red) where the reaction was followed up by GC.

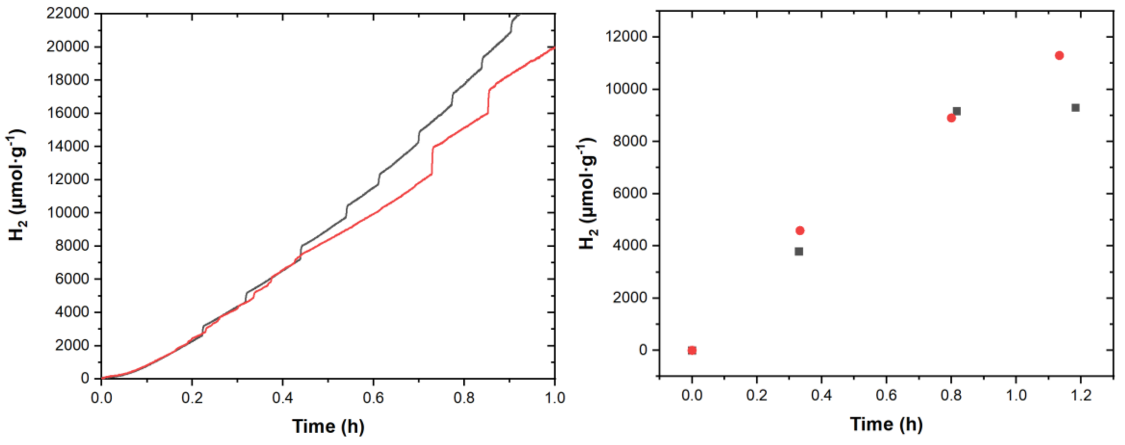

Figure S22. Hydrogen evolution profile of a photocatalytic mixture composed of TpDTz (0.5 mg · mL^{-1}) in a mixture of ascorbic acid/sodium ascorbate buffer 1.6 M and 5 % (w/w) loading of presynthesized PtNPs and irradiated with a 150 W Xe lamp equipped with AM 1.5G filter (100mW · cm^{-2}). **Left)** Two replicates (black and red) where the reaction was followed by Clark sensor. Note that the steps observed in these analyses are due to fast bubble formation that leads to the sudden signal increase when the bubble reaches the sensor located at the headspace of the reactor, see Figure S20. **Right)** Two replicates (black and red) where the reaction was followed up by GC.

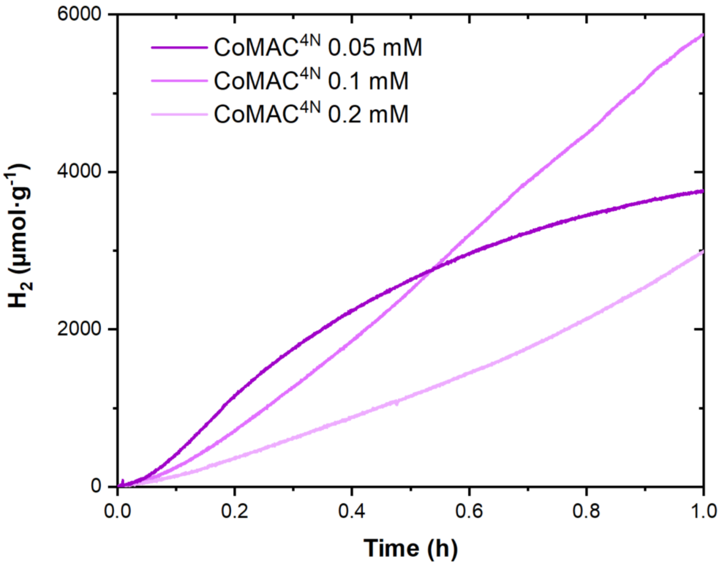

Figure S23. Hydrogen evolution profile of a photocatalytic mixture composed of TpDTz (0.5 mg · mL^{-1}) in a mixture of ascorbic acid/sodium ascorbate buffer 1.6 M and a loading of CoMAC^{4N} of 0.5 % (w/w) (0.05 mM, dark purple), 1 % (w/w) (0.1 mM, purple) and 2 % (w/w) (0.02 mM, light purple) irradiated with a 150 W Xe lamp equipped with AM 1.5G filter (100 mW · cm^{-2}).

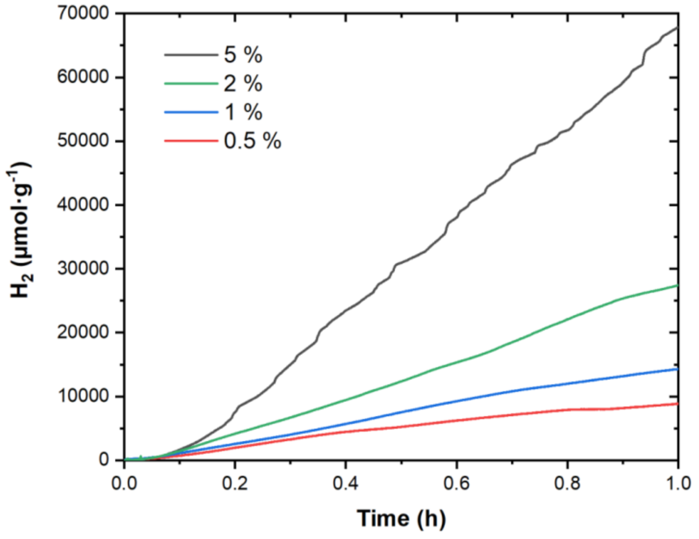

Figure S24. Hydrogen evolution profile of a photocatalytic mixture composed of TpDTz (0.5 mg · mL^{-1}) in a mixture of ascorbic acid/sodium ascorbate buffer 1.6 M and different loading of PtNPs (0.5-5 % w/w) irradiated with a LED light λ_{em} = 525 nm (100 mW · cm^{-2}).

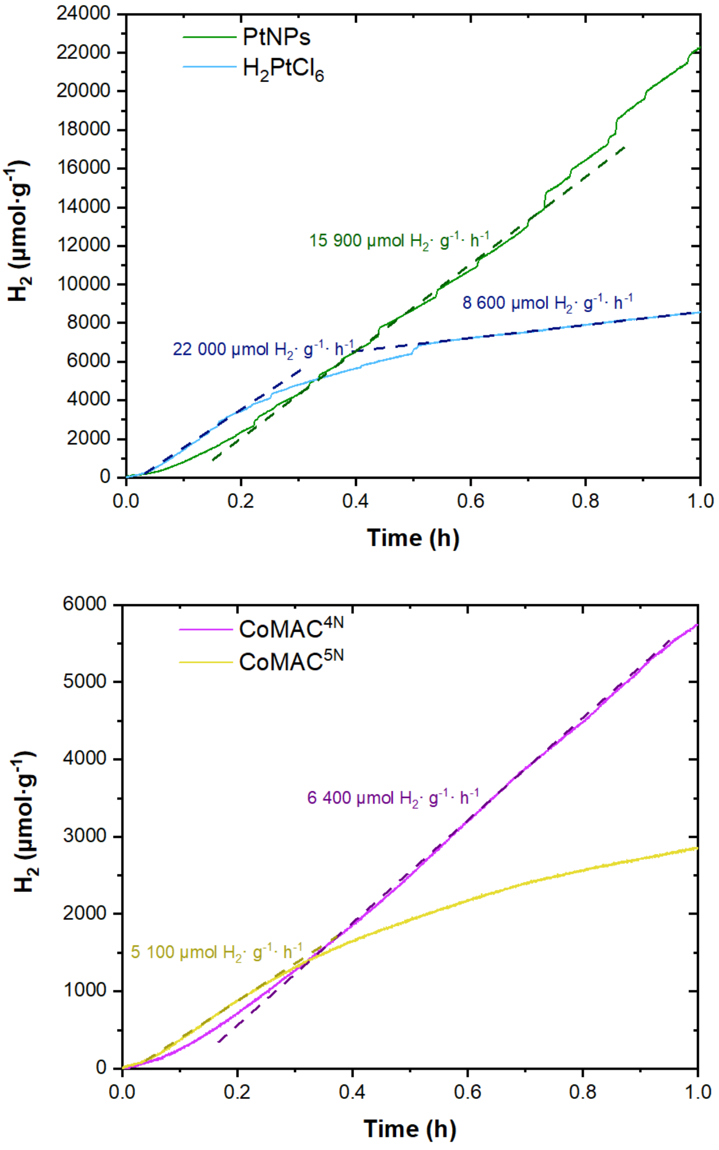

Figure 25. Comparison of light induced hydrogen evolution reaction using TpDTz and different catalytic species in the presence of ascorbic acid:sodium ascorbate (Asc) sacrificial electron donor under white light illumination AM 1.5G (100 mW · cm^{-2}). **Top)** System based on PtNPs (5% Pt w/w) formed *in situ* (blue) or pre-synthesized (green). **Bottom)** System based on cobalt molecular complex (1mM, 1% Co w/w) $CoMAC^{4N}$ (purple) or $CoMAC^{5N}$ (yellow). Dotted lines indicate the slope used to calculate the TOF values indicated in the graph in μmol H_2 · g^{-1} · h^{-1}. A Clark sensor was used to measure hydrogen evolution. Note that the steps observed in the analysis of the Pt catalalysts (top) are due to fast bubble formation that leads to the sudden signal increase when the bubble reaches the sensor located at the headspace of the reactor, see Figure S20.

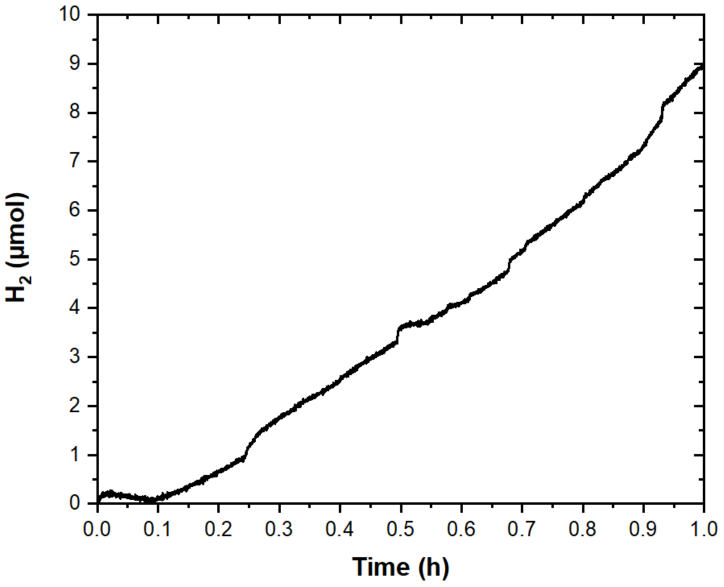

Figure S26. Hydrogen evolution profile of a photocatalytic mixture composed of TpDTz (0.5 mg · mL^{-1}) and PtNPs (5 % w/w) in phosphate buffer pH 7 containing sodium ascorbate (1.6 M), irradiated with a LED light λ_{em} = 525 nm (100 mW · cm^{-2}).

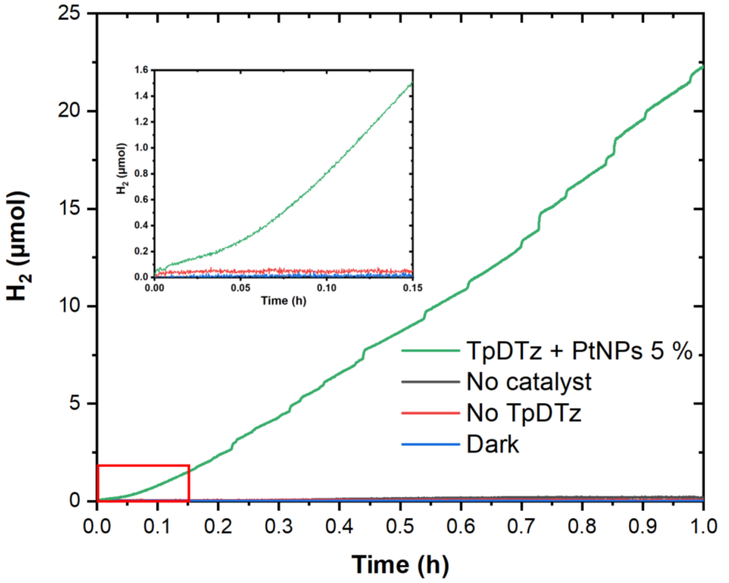

Figure S27. Blank experiments run in the absence of TpDTz 2D-COF (red) or catalyst (black) or light (blue). Standard conditions: TpDTz (0.5 mg · mL^{-1}) in a mixture of ascorbic acid/sodium ascorbate buffer 1.6 M and catalyst PtNPs (5 % w/w) irradiated with white light illumination AM 1.5G (100 mW · cm^{-2}). Green trace corresponds to the full system containing all components.

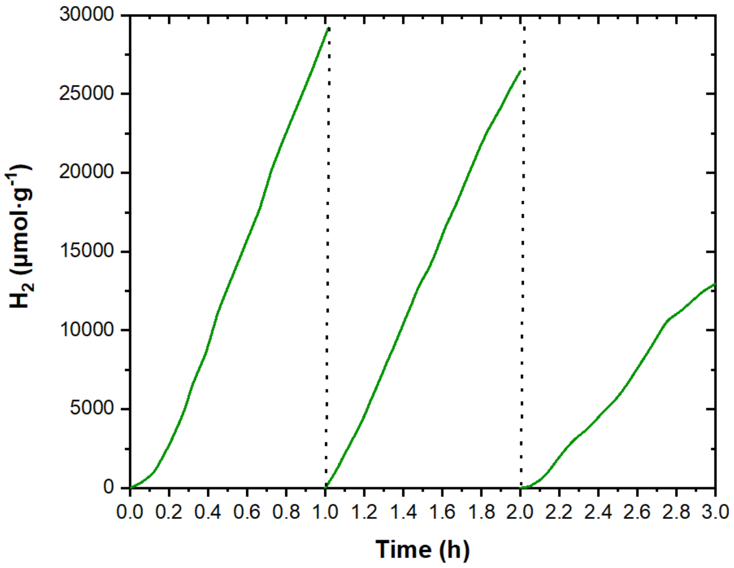

Figure S28. Three consecutive hydrogen evolution photocatalytic cycles using TpDTz (0.5 mg · mL^{-1}) and PtNPs (5 % w/w, batch 2) in a mixture of ascorbic acid/sodium ascorbate buffer 1.6 M, irradiated with a LED light λ_{em} = 525 nm (100 mW · cm^{-2}. After 1 h irradiation, the system was centrifugated to remove the supernatant, then the COF was washed with MeOH x 2 and DCM x 1 using centrifugation and dried at 60 ºC overnight in a vacuum oven. The material was then reused with a new buffer solution. For the consecutive runs no additional PtNPs were added confirming the formation of the PtNPs/TpDTz hybrid material during the first run.

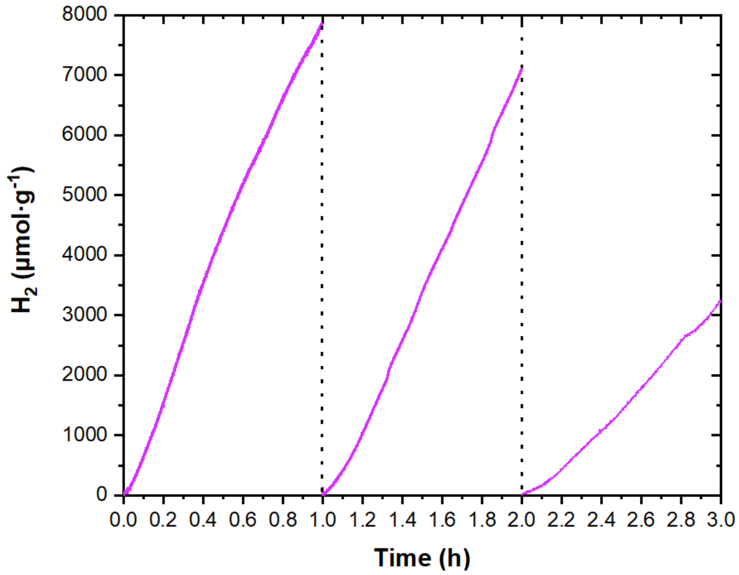

Figure S29. Three consecutive hydrogen evolution photocatalytic cycles using TpDTz (0.5 mg · mL^{-1}) and CoMAC^{4N} (1mM, 1% Co w/w) in a mixture of ascorbic acid/sodium ascorbate buffer 1.6 M, irradiated with a LED light λ_{em} = 525 nm (100 mW · cm^{-2}). After 1 h of irradiation, the system was centrifugated to remove the supernatant, then the COF was washed with MeOH x 2 and DCM x 1 using centrifugation and dried at 60 ºC overnight in a vacuum oven. The material was then reused with a new buffer solution containing additional CoMAC^{4N} (1mM, 1% Co w/w) for the consecutive run.

11. Post-catalysis characterization

a)

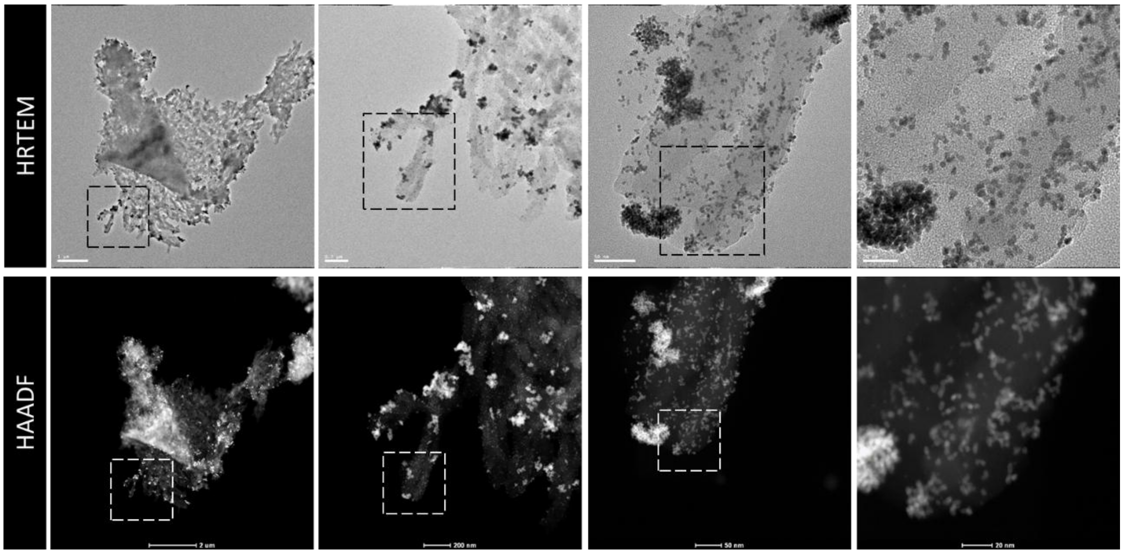

b)

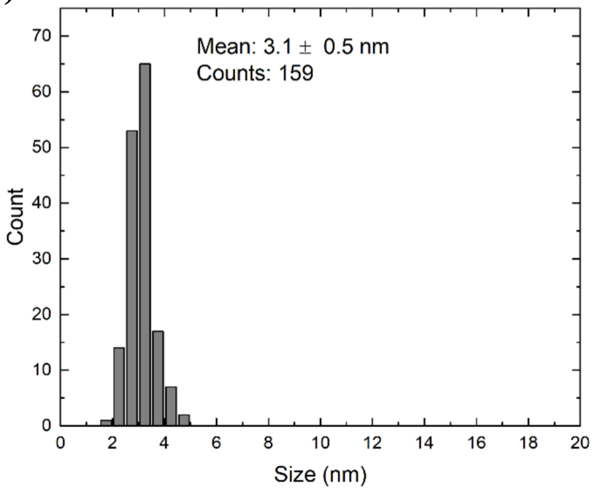

Figure S30. **a)** HRTEM (**top**) and HAADF (**bottom**) at different zooms of TpDTz after 1 h photocatalysis using PtNPs as catalyst resulting in the TpDTz@PtNPs hybrid material, at 2000 nm, 200 nm, 50 nm and 20 nm (left to right respectively). **b)** Size distribution analysis of the PtNPs within the TpDTz@PtNPs hybrid material.

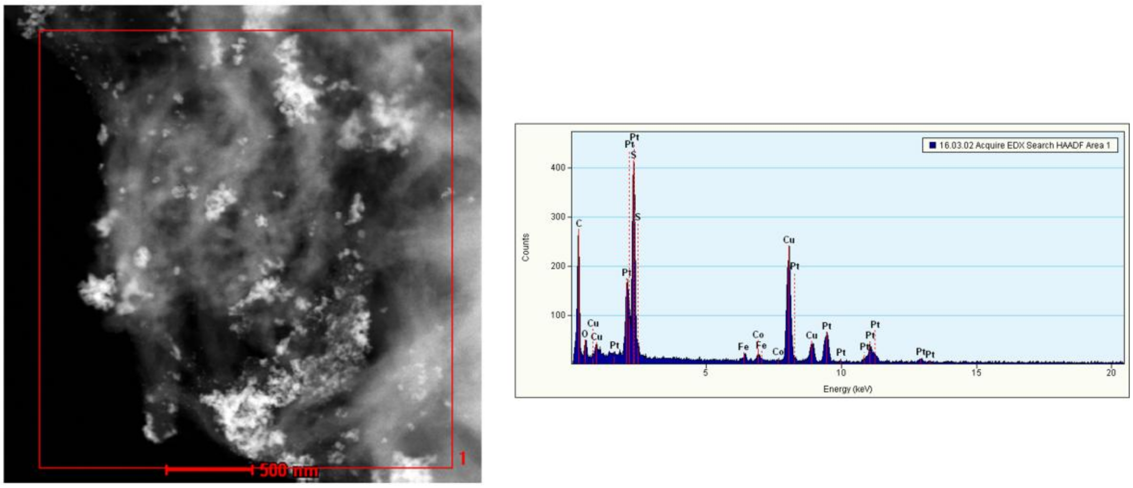

Figure S31. EDX of TpDTz after 1 h photocatalysis using PtNPs as catalyst.

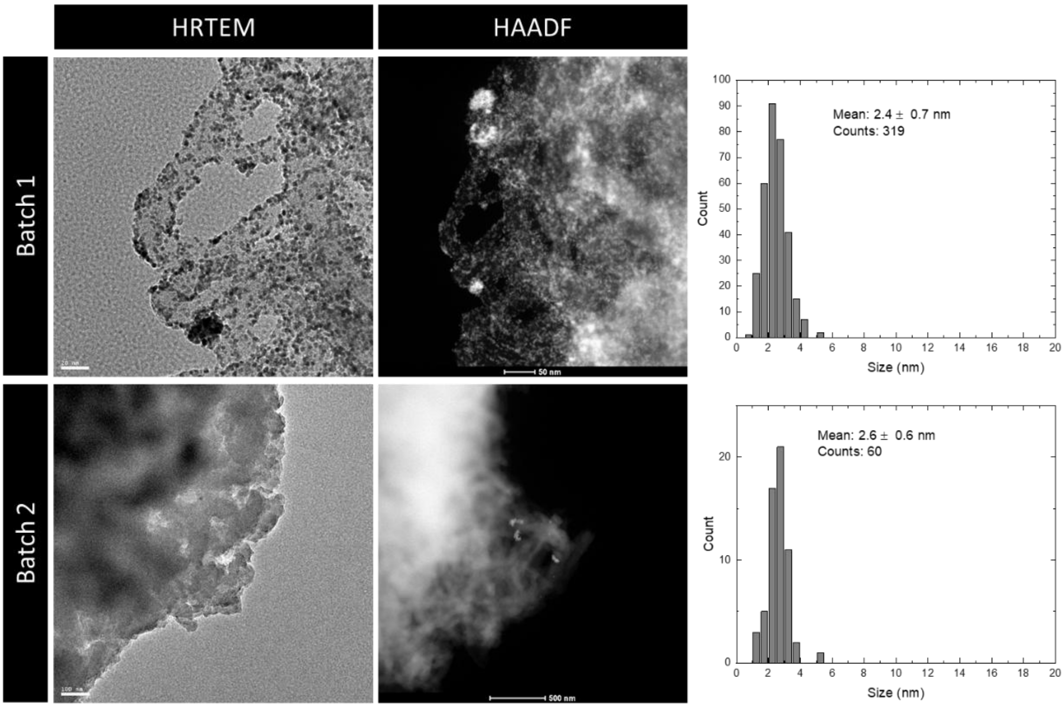

Figure S32. HRTEM, HAADF images and size distribution analyses of two representative batches of TpDTz after 1 h photocatalysis using H_2PtCl_6 as catalyst precursor. Note that for Batch 2, only 60 counts are given due to the low number of nanoparticles observed in this sample. Indeed, the formation of nanoparticles for the experiments involving H_2PtCl_6 was not always reproducible and contained significantly lower number of nanoparticles compared to the preformed PtNPs postcatalysis samples as illustrated in Figures S30-S31.

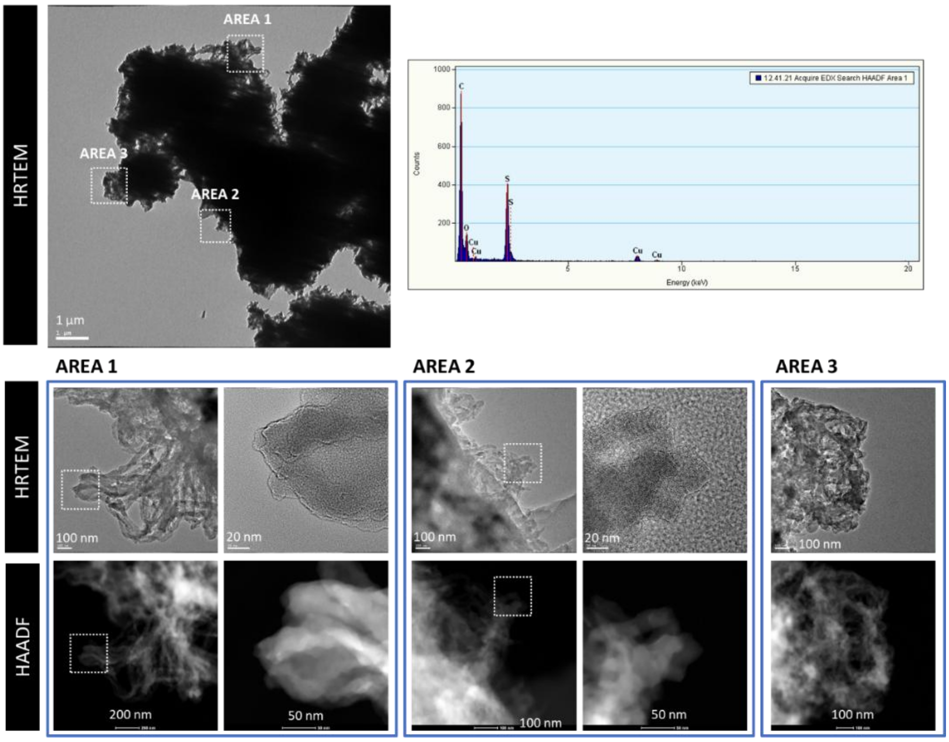

Figure S33. HRTEM, HAADF images and EDX analysis of TpDTz after 1 h photocatalysis using CoMAC^{4N} as catalyst.

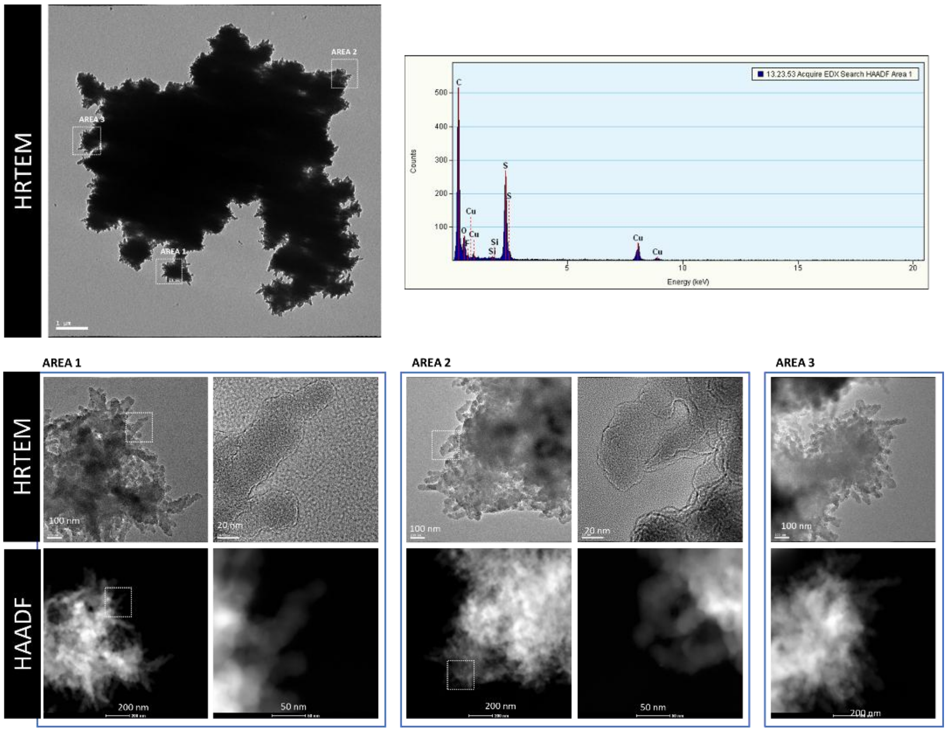

Figure S34. HRTEM, HAADF images and EDX analysis of TpDTz after 1 h photocatalysis using CoMAC^{5N} as catalyst.

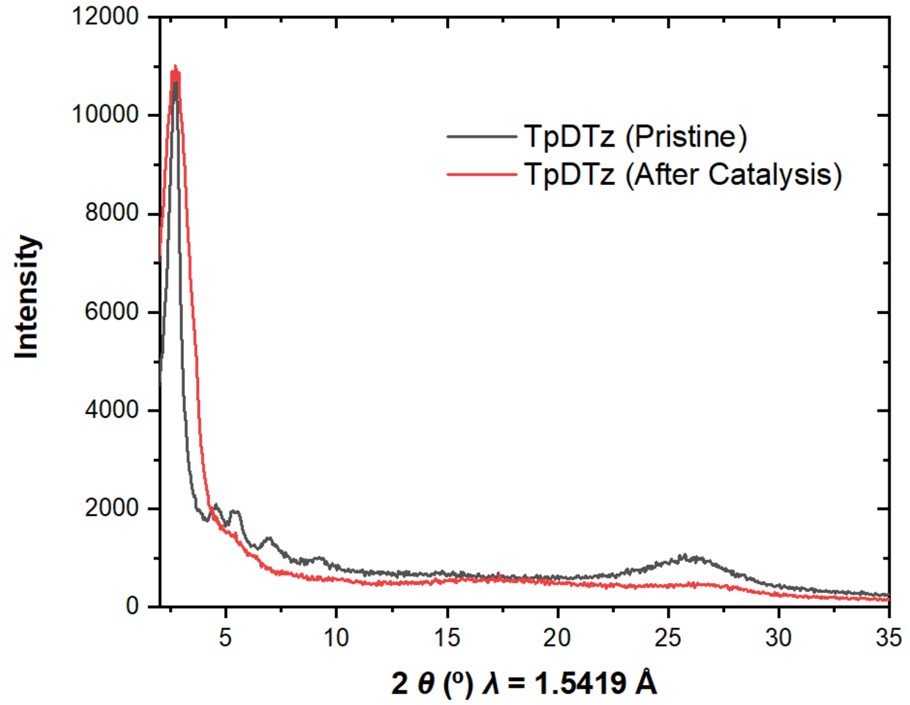

Figure S35. PXRD of TpDTz before (black) and after 2 h photocatalysis using PtNPs as catalyst (red).

12. Estimation of VB and CB of TpDTz

The non-aqueous cyclic voltammetry experiments were conducted by using a modified FTO (working), a platinum mesh (counter) and a SCE (reference) electrodes. The modified FTO electrode was prepared following a similar procedure used in reference 1. The TpDTz COF (2 mg) was suspended in a mixture of deionized water (50 μL), isopropanol (30 μL) and 5 % Nafion-117 solution (20 μL) by sonicating for 30 min. A sample of 100 μL of the resulting suspension were drop-casted on a clean FTO electrode (1 × 1 cm) and dried overnight in an oven at 60 ºC. The three-electrode electrochemical cell was purged with N_2, and the modified FTO electrode was submerged in the electrolyte solution composed of acetonitrile-0.1 M [n-$Bu_4N]_4PF_6$. The reduction onset potential was extracted from the x-intercept of the linear fits in the voltammogram and is consistent with previous results within experimental error of the technique (Figure S36).[1] Then, the following equations were used to calculate the potential of the conductive and valence band of the material *vs* vacuum, the potential of the optical bandgap ($E_{g,opt}$ = 2.12 eV) was extracted from the Kubelka-Munk function in Figure S6.

$$E_{CB} = -(E_{red\ onset\ vs\ SCE} - E_{1/2,Fc} + 5.1)\ eV$$

$$E_{VB} = E_{CB} - E_{g,opt}$$

The resulting energy levels of the material *vs* vacuum level were calculated as E_{CB} = -3.8 and E_{VB} = -5.9 eV.

For the potentials *vs* RHE the following equations were used:

$$E_{CB\ vs\ NHE} = E_{red\ onset\ vs\ SCE} + 0.241\ V$$

$$E_{VB\ vs\ NHE} = E_{CB\ vs\ NHE} + E_{g,opt}$$

The resulting potentials of the materials *vs* NHE were calculated as E_{CB} = -0.7 and E_{VB} = 1.4 V.

	Vacuum (eV)	NHE (V)
E_{CB}	-3.8	-0.7
E_{VB}	-5.9	1.4

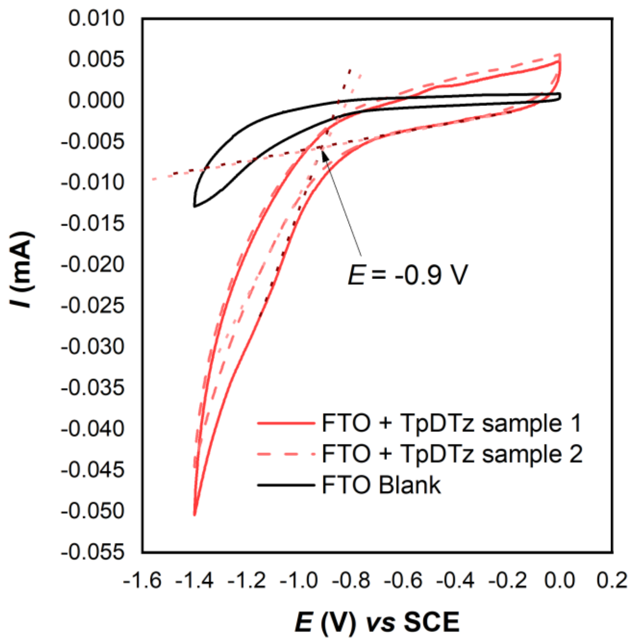

Figure S36. CV analysis of TpDTz deposited on FTO (red) and bare FTO (grey) (WE, A ≈ 1 cm^2) in acetonitrile-0.1 M [n-$Bu_4N]_4PF_6$, CE: platinum mesh, RE: SCE (converted to NHE by adding 0.241 V).

13.Comparative of relevant HER photocatalytic systems

Table S2. Selected HER photocatalytic systems based on 2D-COF and other relevant organic materials.

2D-COF	Catalytic Center	SED	Activity (μmol $h^{-1}g^{-1}$)	Year[ref]
TpDTz	Ni-ME cluster 2 % (w/w)	TEOA 10 % (v/v)	941 (300 W Xe light, 100 mW·cm^{-2}, λ > 420 nm)	2019[1]
NiS/TpBD-COF	NiS 3 % (w/w)	Na_2S/Na_2SO_3 0.1 M and 0.1 M	3 840 (300 W Xe light, λ > 420 nm)	2022[6]
COF-AD1	cobaloxime	TEOA 1 % (v/v)	7 072 (450 W Xe light, 1.95 W·cm^{-2}, λ > 420 nm)	2022[7]
COF-OH-3	Pt 1 % (w/w)	Ascorbic acid 0.1 M	9 890 (300 W Xe light, λ = 420 nm)	2022[8]
TpBpy-Cu/Co	Cu 3.45 % (w/w) Co 1.77 % (w/w)	BIH 0.13 M, AcOH/DMF 0.015 % (v/v)	12 160 (300 W Xe light, 520 mW·cm^{-2} λ > 420 nm)	2025[9]
NKCOF-113-M	Pt 5 % (w/w)	TEOA 10 % (v/v)	13 100 (300 W Xe light, λ > 420 nm)	2022[10]
FS-COF	Pt 8 % (w/w)	Ascorbic acid 0.1 M	10 100 (300 W Xe light, λ > 420 nm)	2018[11]
FS-COF+ WS5F			16 300 (300 W Xe light, λ > 420 nm)	
Tp-COF/TiO_2	Pt 6 % (w/w)	Ascorbic acid 0.05 M	25 900 (300 W Xe light, λ > 420 nm)	2022[12]
COF-JLU35	Pt 1 % (w/w)	Ascorbic acid 0.1 M	70 800 (300 W Xe light, 180 mW·cm^{-2}, λ > 420 nm)	2023[13]
TpPa-Cl_2	Pt 3 % (w/w)	Ascorbic acid 0.1 M	99 226 (300 W Xe light, λ > 420 nm)	2024[14]

COF-JLU100	Pt 12 % (w/w)	TEOA 10 % (v/v)	107 380 (300 W Xe light, 100 mW·cm^{-2}, λ > 420 nm)	2022[15]
TpPa-SCOF-An	Pt 0.7 % (w/w)	Ascorbic acid 0.1 M	126 000 (300 W Xe light, λ > 420 nm)	2025[16]
IMDA-AA	Pt 1.5 % (w/w)	Ascorbic acid 0.1 M	171 210 (300 W Xe light, 60 mW·cm^{-2}, λ > 420 nm)	2025[17]
COF-954	PtNPs 5% (w/w)	Ascorbic acid 0.1 M	137 230 (300 W Xe light, λ > 420 nm)<br>191 700 (300 W Xe light, λ > 350 nm)	2024[18]
FOOCOF-PDI	Pt 2 % (w/w)	Ascorbic acid 0.1 M	265 000 (300 W Xe light, 160 mW·cm^{-2}, λ > 420 nm)	2025[19]
Ni-COF-SCAU-1	Pt 2 % (w/w)	Ascorbic acid 0.1 M	197 460 (350 W Xe light, λ > 420 nm)<br>272 220 (350 W Xe light, λ > 300 nm)	2023[20]
COF-JLU45	Pt 1 % (w/w)	Ascorbic acid 0.1 M	272 500 (300 W Xe light, λ > 420 nm)	2025[21]
TFP-BpyD nano-COF	Pt 15% (w/w)	Ascorbic acid 0.2 M	392 000 (300 W Xe light, λ > 420 nm)	2024[22]
TpDTz	PtNPs 5% (w/w)	Sodium ascorbate Ascorbic acid 1:1	15 900 (AM 1.5G, 100 mW·cm^{-2})<br>106 000 (LED 525 nm, 100 mW·cm^{-2})	Tw[a]
TpDTz	PtNPs 1% (w/w)	Sodium ascorbate Ascorbic acid 1:1	28 300 (LED 525 nm, 100 mW·cm^{-2})	Tw[a]
TpDTz	CoMAC^{4N} 1% Co (w/w)	Sodium ascorbate Ascorbic acid 1:1	6 400 (AM 1.5G)<br>10 400 (LED 525 nm, 100 mW·cm^{-2})	Tw[a]

Organic material	Catalytic Center	SED	Activity (μmol $h^{-1}g^{-1}$)	Year[ref]
p-CN/Ni-COF	Ni	TEOA 10 % (v/v)	2 290 (300 W Xe light, 100 mW·cm^{-2}, λ > 420 nm)	2023[23]
C_3N_4	Pt 0.2 % (w/w)	TEOA 10 % (v/v)	2 800 (Nine 5 W white LED, 7.70 mW·cm^{-2}, λ > 420 nm)	2019[24]
COF (12% w/w)/CN composite	Pt 3 % (w/w)	TEOA 10 % (v/v)	13 392 (300 W Xe light, λ > 400 nm)	2023[25]
C_3N_4 (CN-D_{2mg})	Pt 3 % (w/w)	TEOA 10 % (v/v)	14 581 (300 W Xe light, λ > 420 nm)	2024[26]
PTB7-Th/EH-IDTBR	Pt 5 % (w/w)	Ascorbic acid 0.2 M	64 426 (300 W Xe Light, λ > 350 nm)	2020[27]
SA-DADK-H^+	Pt 4 % (w/w)	TEOA 10 % (v/v)	153 100 (300 W Xe Light, λ > 420 nm) 278 200 (300 W Xe Light, λ > 300 nm)	2024[28]
HOF-H_4TBAPy	Pt 5 % (w/w)	Ascorbic acid 0.1 M	358 000 (300 W Xe Light, 100 mW·cm^{-2}, λ > 350 nm) 195 000 (300 W Xe Light, 100 mW·cm^{-2}, λ > 420 nm) 114 000 (solar simulator 100 mW·cm^{-2})	2023[29]
HOF/COF	Pt 5 % (w/w)	Ascorbic acid 0.1 M	390 680 (350 W Xe Light, λ > 420 nm)	2025[30]

[a]Tw = this work.

14.NMR Spectra

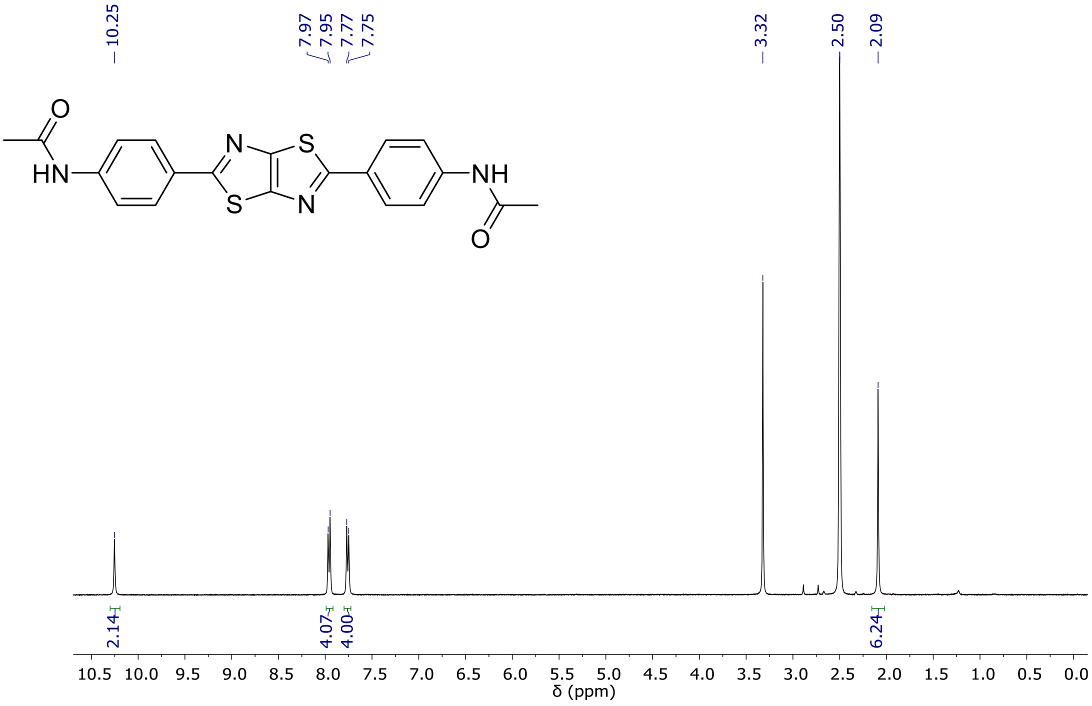

^{1}H NMR (400 MHz, DMSO-d_6) of compound *N*,*N'*-(thiazolo[5,4-*d*]thiazole-2,5-diylbis(4,1-phenylene))diacetamide

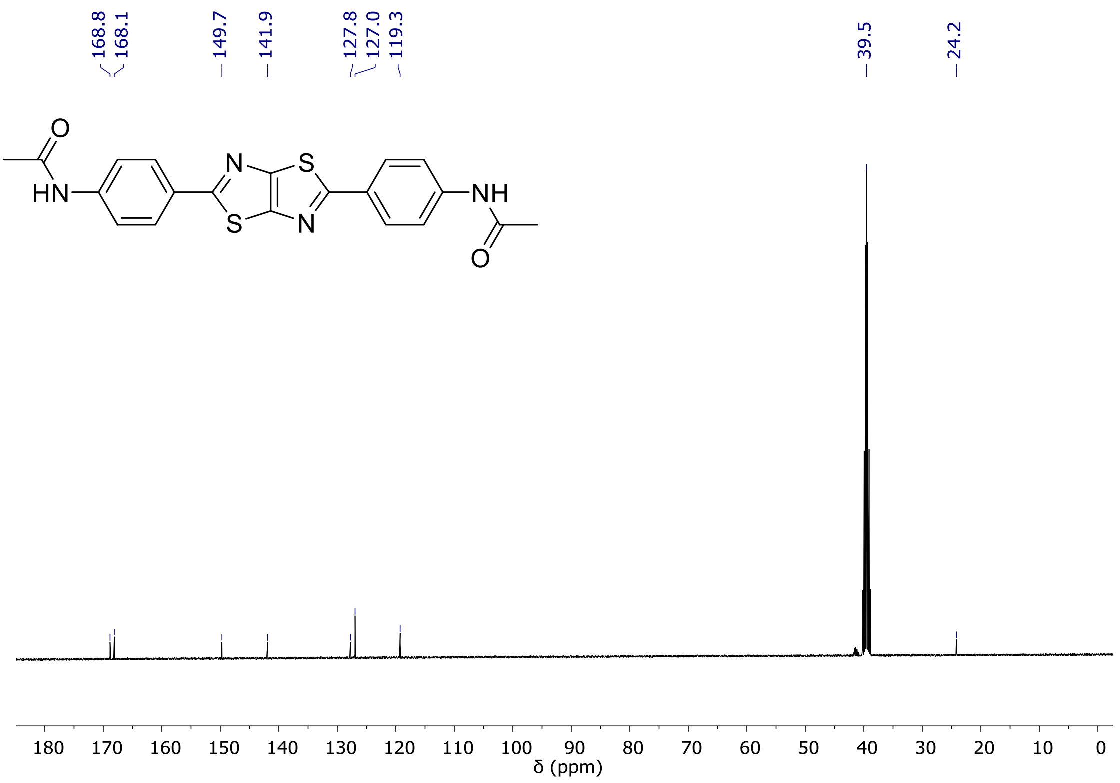

^{13}C {^{1}H} NMR (101 MHz, DMSO-d_6) of compound *N*,*N'*-(thiazolo[5,4-*d*]thiazole-2,5-diylbis(4,1-phenylene))diacetamide

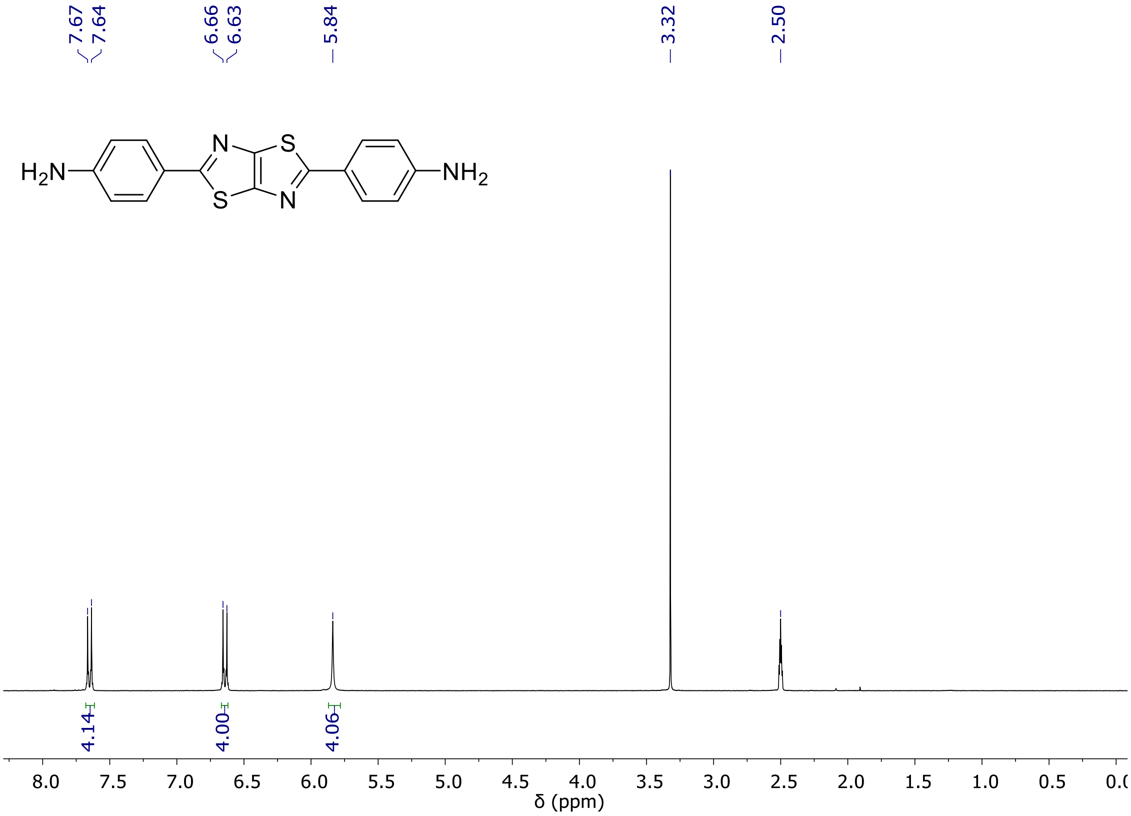

^{1}H NMR (300 MHz, DMSO-d_6) of compound **DTz**

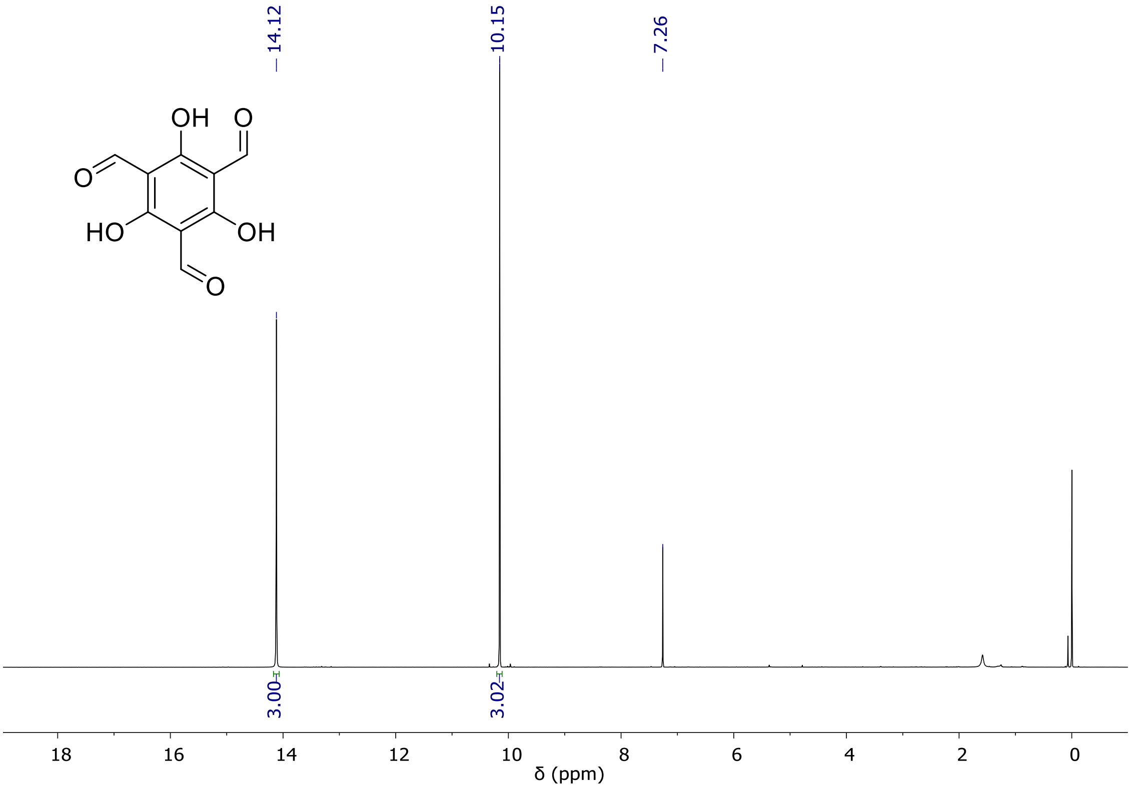

^{1}H NMR (500 MHz, $CDCl_3$) of Tp

15. References

[1] Biswal, B. P.; Vignolo-González, H. A.; Banerjee, T.; Grunenberg, L.; Savasci, G.; Gottschling, K.; Nuss, J.; Ochsenfeld, C.; Lotsch, B. V. Sustained Solar H2 Evolution from a Thiazolo[5,4-*d*]Thiazole-Bridged Covalent Organic Framework and Nickel-Thiolate Cluster in Water. *J. Am. Chem. Soc.* **2019**, *141*, 11082-11092.

[2] Gimbert-Suriñach, C.; Albero, J.; Stoll, T.; Fortage, J.; Collomb, M.-N.; Deronzier, A.; Palomares, E.; Llobet, A. Efficient and Limiting Reactions in Aqueous Light-Induced Hydrogen Evolution Systems Using Molecular Catalysts and Quantum Dots. *J. Am. Chem. Soc.* **2014**, *136*, 7655-7661.

[3] Moonshiram, D.; Gimbert-Suriñach, C.; Guda, A.; Picon, A.; Lehmann, C. S.; Zhang, X.; Doumy, G.; March, A. M.; Benet-Buchholz, J.; Soldatov, A.; Llobet, A.; Southworth, S. H. Tracking the Structural and Electronic Configurations of a Cobalt Proton Reduction Catalyst in Water. *J. Am. Chem. Soc.* **2016**, *138*, 10586-10596.

[4] Grau, S.; Schilling, M.; Moonshiram, D.; Benet-Buchholz, J.; Luber, S.; Llobet, A.; Gimbert-Suriñach, C. Electrochemically and Photochemically Induced Hydrogen Evolution Catalysis with Cobalt Tetraazamacrocycles Occurs through Different Pathways. *ChemSusChem* **2020**, *13*, 2745-2752.

[5] Willery, M.; Julliard, P.-G.; Molton, F.; Thomas, F.; Fortage, J.; Costentin, C.; Collomb, M.-N. Mechanism of Electrochemical Proton Reduction Catalyzed by a Cobalt Tetraaza Schiff Base Macrocyclic Complex: Ligand Protonation and/or Influence of the Chloro Ligand. *ACS Catal.* **2024**, *14*, 11352-11365.

[6] Zhang, H.; Lin, Z.; Guo, J. Enhanced Photocatalytic H2 Evolution over Covalent Organic Frameworks through an Assembled NiS Cocatalyst. *RSC Adv.* **2022**, *12*, 14932–14938.

[7] Aand, D.; Sk, S.; Kumar, K.; Pal, U.; Singh, A. K. Boosting Photocatalytic Hydrogen Generation by the Combination of Tunable Cobaloxime and Covalent Organic Framework. *Int. J. Hydrogen Energy* **2022**, *47*, 7180-7188.

[8] Chen, Y.; Luo, X.; Zhang, J.; Hu, L.; Xu, T.; Li, W.; Chen, L.; Shen, M.; Ren, S.-B.; Han, D.-M.; Ning, G.-H.; Li, D. Bandgap Engineering of Covalent Organic Frameworks for Boosting Photocatalytic Hydrogen Evolution from Water. *J. Mater. Chem. A Mater. Energy Sustain.* **2022**, *10*, 24620-24627.

[9] Shao, M.; Wang, A.; Peng, J.; Song, X.; Wang, L. Dual Non-Noble-Metal-Immobilized Covalent Organic Frameworks for Visible-Light-Driven Photocatalytic Hydrogen Evolution. *Inorg. Chem. Front.* **2025**.

[10] Zhao, Z.; Chen, X.; Li, B.; Zhao, S.; Niu, L.; Zhang, Z.; Chen, Y. Spatial Regulation of Acceptor Units in Olefin-linked COFs toward Highly Efficient Photocatalytic H2 Evolution. *Adv. Sci. (Weinh.)* **2022**, *9*, 2203832.

[11] Wang, X.; Chen, L.; Chong, S. Y.; Little, M. A.; Wu, Y.; Zhu, W.-H.; Clowes, R.; Yan, Y.; Zwijnenburg, M. A.; Sprick, R. S.; Cooper, A. I. Sulfone-Containing Covalent Organic Frameworks for Photocatalytic Hydrogen Evolution from Water. *Nat. Chem.* **2018**, *10*, 1180-1189.

[12] Zhang, W.; Wang, S.; Wen, N.; Zhao, J.; Guo, W.; Wu, S.; Zhang, P.; Lin, Q.; Xu, J.; Long, J. TiO2-Promoted Electron-Tunneling of COF-Based MIS Nanostructures for Efficient Photocatalytic Hydrogen Production. *Mater. Today Chem.* **2022**, *26*, 101150.

[13] Li, Z.; Deng, T.; Ma, S.; Zhang, Z.; Wu, G.; Wang, J.; Li, Q.; Xia, H.; Yang, S.-W.; Liu, X. Three-Component Donor−π–Acceptor Covalent–Organic Frameworks for Boosting Photocatalytic Hydrogen Evolution. *J. Am. Chem. Soc.* **2023**, *145*, 8364-8374.

[14] Jiang, S.; Niu, H.; Sun, Q.; Zhao, R.; Li, N.; Cai, Y. Significant improvement of photocatalytic hydrogen evolution performance in covalent organic frameworks: substituent fine-tuning. *J. Mater. Chem. A* **2024**, *12*, 11416-11423.

[15] Ma, S.; Deng, T.; Li, Z.; Zhang, Z.; Jia, J.; Li, Q.; Wu, G.; Xia, H.; Yang, S.-W.; Liu, X. Photocatalytic Hydrogen Production on a Sp2-carbon-linked Covalent Organic Framework. *Angew. Chem. Int. Ed Engl.* **2022**, *61,* e202208919.

[16] Lin, Z.; Yu, X.; Zhao, Z.; Ding, N.; Wang, C.; Hu, K.; Zhu, Y.; Guo, J. Controlling crystallization in covalent organic frameworks to facilitate photocatalytic hydrogen production. *Nat. Commun.* **2025**, *16*, 1940.

[17] Shuang, Y.; Zhang, Y.; Wang, H.; Li, L.; Hao, X.; Ma, Z.; Wang, S.; Wang, J.; Wang, F.; Yang, X.; Guo, P.; Xu, F.; Wang, H.; Ye, Q.; Liu, W.; Jian, J.; Wang, H. Proton-Mediated Topological Interlayer Shift in 2D Covalent Organic Frameworks for Efficient Photocatalysis. *Adv. Mater.* **2025**, *37*, e2500468.

[18] Zhong, Y.; Dong, W.; Ren, S.; Li, L. Oligo(Phenylenevinylene)-Based Covalent Organic Frameworks with Kagome Lattice for Boosting Photocatalytic Hydrogen Evolution. *Adv. Mater.* **2024**, *36*, e2308251.

[19] Shen, R.; Huang, C.; Hao, L.; Liang, G.; Zhang, P.; Yue, Q.; Li, X. Ground-state charge transfer in single-molecule junctions covalent organic frameworks for boosting photocatalytic hydrogen evolution. *Nat. Commun.* **2025**, *16*, 2457.

[20] Shen, R.; Li, X.; Qin, C.; Zhang, P.; Li, X. Efficient Photocatalytic Hydrogen Evolution by Modulating Excitonic Effects in Ni-intercalated Covalent Organic Frameworks. *Adv. Energy Mater.* **2023**, *13*, 2203695.

[21] Ma, S.; Li, Z.; Hou, Y.; Li, J.; Zhang, Z.; Deng, T.; Wu, G.; Wang, R.; Yang, S.-w.; Liu, S. Fully Conjugated Benzobisoxazole-Bridged Covalent Organic Frameworks for Boosting Photocatalytic Hydrogen Evolution. *Angew. Chem. Int. Ed.* **2025**, *64*, e202501869.

[22] Zhao, W.; Luo, L.; Cong, M.; Liu, X.; Zhang, Z.; Bahri, M.; Li, B.; Yang, J.; Yu, M.; Liu, L.; Xia, Y.; Browning, N. D.; Zhu, W.-H.; Zhang, W.; Cooper, A. I. Nanoscale covalent organic frameworks for enhanced photocatalytic hydrogen production. *Nat. Commun.* **2024**, *15*, 6482.

[23] Zhu, L.; Liang, Z.; Li, H.; Xu, Q.; Jiang, D.; Du, H.; Zhu, C.; Li, H.; Lu, Z.; Yuan, Y. A π-Conjugated van Der Waals Heterostructure between Single-Atom Ni-Anchored Salphen-Based Covalent Organic Framework and Polymeric Carbon Nitride for High-Efficiency Interfacial Charge Separation. *Small* **2023**, *19*, e2301017.

[24] Li, Z.; Zhou, S.; Yang, Q.; Zhang, Z.; Fang, X. Insight into the Enhanced Hydrogen Evolution Activity of 2,4-Diaminopyrimidine-Doped Graphitic Carbon Nitride Photocatalysts. *J. Phys. Chem. C Nanomater. Interfaces* **2019**, *123*, 2228–2237.

[25] Liu, Y.; Jiang, L.; Tian, Y.; Xu, Z.; Wang, W.; Qiu, M.; Wang, H.; Li, X.; Zhu, G.; Wang, Y. Covalent Organic Framework/g-C3N4 van Der Waals Heterojunction toward H2 Production. *Inorg. Chem.* **2023**, *62*, 3271-3277.

[26] Zhou, F.; Chen, X.; Zhao, Y.; Cheng, J.; Xu, G. A Novel Graphitic Carbon Nitride Photocatalyst with Ultramicro Amounts of Intramolecular Donor-Acceptor Structures for Excellent Photocatalytic Hydrogen Production. *J. Photochem. Photobiol. A Chem.* **2024**, *449*, 115378.

[27] Kosco, J.; Bidwell, M.; Cha, H.; Martin, T.; Howells, C. T.; Sachs, M.; Anjum, D. H.; Gonzalez Lopez, S.; Zou, L.; Wadsworth, A.; Zhang, W.; Zhang, L.; Tellam, J.; Sougrat, R.; Laquai, F.; DeLongchamp, D. M.; Durrant, J. R.; McCulloch, I. Enhanced Photocatalytic Hydrogen Evolution from Organic Semiconductor Heterojunction Nanoparticles. *Nat. Mater.* **2020**, *19*, 559–565.

[28] Zhu, X.; Jia, Y.; Liu, Y.; Xu, J.; He, H.; Wang, S.; Shao, Y.; Zhai, Y.; Zhu, Y. Enhancing Built-in Electric Fields via Molecular Symmetry Modulation in Supramolecular Photocatalysts for Highly Efficient Photocatalytic Hydrogen Evolution. *Angew. Chem. Int. Ed Engl.* **2024**, *63*, e202405962.

[29] Zhou, Q.; Guo, Y.; Zhu, Y. Photocatalytic Sacrificial H2 Evolution Dominated by Micropore-Confined Exciton Transfer in Hydrogen-Bonded Organic Frameworks. *Nat. Catal.* **2023**, *6*, 574–584.

[30] Gao, R.; Shen, R.; Huang, C.; Huang, K.; Liang, G.; Zhang, P.; Li, X. 2D/2D Hydrogen-Bonded Organic Frameworks/Covalent Organic Frameworks S-Scheme Heterojunctions for Photocatalytic Hydrogen Evolution. *Angew. Chem. Int. Ed Engl.* **2025**, *64*, e202414229.
